# Roadmap to Challenges in Limited Cellularity Specimens from Pancreatic Neuroendocrine Neoplasms: Diagnostic Tools for the Most Appropriate Management of Limited Material

**DOI:** 10.1007/s12022-025-09870-3

**Published:** 2025-07-16

**Authors:** Stefano La Rosa, Roberta Maragliano, Deborah Marchiori

**Affiliations:** 1 Unit of Pathology, Department of Medicine and Technological Innovation, University of Insubria, Viale Borri 57, 21100 Varese, Italy; 2 Hereditary Cancer Research Center, Department of Medicine and Technological Innovation, University of Insubria, Varese, Italy; 3 Unit of Pathology, Department of Oncology, ASST Sette Laghi, Varese, Italy

**Keywords:** Fine needle aspiration, Fine needle biopsy, Neuroendocrine neoplasms

## Abstract

Fine needle aspiration (FNA) and fine needle biopsy (FNB) procedures are increasingly employed in the diagnostic work-up of pancreatic masses. These procedures represent a challenge for pathologists who have to adapt to handling specimens of limited cellularity. In several cases, FNA and FNB specimens are the only available material, as many pancreatic neoplasms are surgically unresectable at the time of the initial diagnosis. In the present review paper, the diagnosis of pancreatic neuroendocrine neoplasms in limited cellularity specimens is presented using a morphology-based approach. The aim is to provide a practical guide for pathologists to select the most appropriate ancillary techniques to be used for the diagnostic work-up, while conserving precious material. The integration of morphology, immunohistochemistry, and molecular biology will be discussed to provide the reader with practical tools to solve the main differential diagnostic problems encountered in routine practice when working with cytological samples or small biopsies.

## Introduction

In the era of interventional radiology, most pancreatic masses are diagnosed using fine needle aspiration (FNA) or fine needle biopsy (FNB). In many cases, these specimens represent the only available material because pancreatic neoplasms are often surgically unresectable at the time of the initial diagnosis. Consequently, pathologists need to become increasingly aware and proficient in the handling of limited cellularity specimens.

Morphological examination, which is the first step of this diagnostic process, often needs to be implemented with ancillary analyses to resolve specific differential diagnoses. Therefore, well-defined criteria are necessary for the selection of the appropriate sequence of immunohistochemical stains to ensure the correct diagnosis while conserving precious material. In most cases, molecular pathology does not play a crucial role in this process, since the molecular background is typically not diagnostic, with a few exceptions.

The diagnostic approach to pancreatic masses (FNA versus FNB) depends on local practices and the expertise of local endoscopists and pathologists. For this reason, some centers prefer cytology while others routinely employ a histological approach using FNB.

To cover both approaches and to assist readers in addressing specific differential diagnostic issues, this review paper is organized into three sections. The first section outlines the technical aspects related to the preparation of adequate FNA or FNB specimens. The second section presents the diagnostic criteria for the cytological diagnosis of both pancreatic neuroendocrine tumors (panNETs) and pancreatic neuroendocrine carcinomas (panNECs), followed by a discussion of the key cytological features of related mimickers. The third section focuses on the histological diagnosis of panNETs and panNECs in FNB specimens.

The integration of morphology with immunohistochemistry and, when necessary, with molecular biology will allow us to discuss the five challenges encountered in the diagnosis of pancreatic neuroendocrine neoplasms (panNENs) in FNB preparations: (I) grading of panNETs; (II) distinguishing panNETs from panNECs; (III) distinguishing panNETs from non-neuroendocrine mimickers; (IV) distinguishing small cell panNECs from non-neuroendocrine mimickers; and (V) distinguishing large cell panNECs from non-neuroendocrine mimickers.

## Technical Aspects for the Preparation of FNA or FNB Specimens

Endoscopic ultrasound (EUS)-guided FNA or FNB are increasingly used in the diagnostic work-up of pancreatic masses. When performed by experts, these procedures are both safe and cost-effective [1, 2]. EUS-FNA can be combined with the cytological rapid on-site evaluation (ROSE) to increase the yield of the collected material and to optimize triage [3]. The first pass is used to prepare a smear that is rapidly stained with toluidine-blue (approximately 10 s) to assess the sample quality and to provide a preliminary diagnosis. If possible, one or two additional passes are recommended to increase the cellular yield to produce a cell block, which allows for the subsequent testing of multiple immunocytochemical or molecular markers.

Recently, after the introduction of the latest biopsy devices, the benefits of ROSE have been questioned, since some studies have shown that its use does not significantly improve specimen adequacy. In high-volume centers with experienced endoscopists, ROSE does not provide a significant benefit although it may offer a benefit in centers with less experienced endoscopists [2, 4].

In cases with prior or concurrent indeterminate FNA, FNB has demonstrated slightly better diagnostic performance, providing more cellular tissue samples and more material for ancillary studies than matched FNAs [5]. Furthermore, FNB specimens have the advantage of providing *d’emblée* adequate histological tissue cores which can be useful when ancillary techniques are required. Comparative studies have shown that EUS-FNB alone is superior to EUS-FNA with ROSE [6–8]. However, the choice of the fine needle procedure strongly depends on the expertise of the endoscopist and pathologist, as well as local practices and resources [9]. FNB specimens are processed following standard protocols for histological slide preparation.

Regardless of the procedure used (FNA or FNB), detailed clinical and procedural information is essential to guide pathologists. This includes data on tumor size, location within the pancreas, tissue traversed during FNA/FNB (stomach or duodenum), solid or cystic nature of the lesion, and the presence or absence of a bile duct or pancreatic duct stricture. Given the scarcity of diagnostic material, immunohistochemical or molecular tests need to be carefully selected [9]. It is extremely important that the suspicion of a possible non-pancreatic tumor (i.e., metastasis or lymphoma) should be stated to avoid losing precious material in performing non-pertinent immunohistochemical stains or molecular tests [2].

## Cytological Features and Reporting FNA Specimens

### Cytological Diagnostic Categories

With the aim of providing clear information to better guide the diagnostic approach to pancreaticobiliary disease and to improve patient care, the IARC/WHO has recently developed a comprehensive seven-tiered system for pancreatic cytology. This standardized framework is designed to help pathologists in the interpretation of cytopathological samples, paralleling the WHO classification of pancreatic tumors [10] (Table 1).

**Table 1:** IARC/WHO reporting system for pancreaticobiliary cytopathology

N°	Category name	Entities
1	Inadequate/insufficient/nondiagnostic	
2	Benign/negative for malignancy	Nonneoplastic and neoplastic entities (e.g., inflammatory processes, nonneoplastic mass‐forming lesions, serous cystadenoma)
3	Atypical	
4	Pancreatobiliary neoplasm: low‐risk/grade	Low‐grade MCN Low‐grade IPMN Low‐grade panIN and BilIN Spindle cell lesions, not further classified
5	Pancreatobiliary neoplasm: high risk/grade	High‐grade MCN High‐grade IPMN IOPN ITPN High‐grade panIN High-grade BilIN
6	Suspicious (for malignancy)	
7	Malignant	PDAC, CC, ACC panNET, panNEC, SPN, PB, other

*ACC*, acinar cell carcinoma; *BilIN*, biliary intraepithelial neoplasm; *CC*, cholangiocarcinoma; *IOPN*, intraductal oncocytic papillary neoplasm; *IPMN*, intraductal papillary mucinous neoplasm; *ITPN*, intraductal tubulopapillary neoplasm; *MCN*, mucinous cystic neoplasm; *panIN*, pancreatic intraepithelial neoplasm; *panNEC*, pancreatic neuroendocrine carcinoma; *panNET*, pancreatic neuroendocrine tumor; *PB*, pancreatoblastoma; *PDAC*, pancreatic ductal adenocarcinoma; *PSC,* Papanicolaou Society of Cytopathology; *SPN*, solid pseudopapillary neoplasm; *WHO*, World Health Organization [modified from ref 10]

*Category 1* includes three terms (insufficient/inadequate/nondiagnostic) and refers to samples that do not allow diagnosis due to qualitative or quantitative reasons, resulting from technical, sampling, or preparation issues. Indeed, FNA specimens presenting preparation artifacts, incorrect smearing technique, poor fixation, or abundant blood often lack sufficient diagnostic material, precluding a diagnosis. Moreover, FNAs containing only gastric or duodenal contaminants or normal pancreatic cells in the setting of a radiologically well-defined mass represent a sampling error and are categorized as inadequate as well. In this category, there is no minimum required epithelial cellularity for adequacy. Conversely, the presence of any cellular atypia precludes categorization as Category 1.

*Category 2* includes two terms (benign/negative for malignancy) and refers to samples with unequivocal benign cytopathological features or definite absence of malignancy in adequate cellularity.

*Category 3* (atypical) is used when cells display cytoplasmic, nuclear, or architectural atypia inconsistent with normal or reactive cellular changes, which, however, are quantitatively or qualitatively insufficient to classify the lesion as neoplastic. The pathologist’s experience plays a crucial role in the definition of this category. Overuse of this category should be avoided since it may lead to unnecessary repetition of FNA.

*Category 4* (pancreatobiliary neoplasm low risk/grade) includes features of an intraductal and/or cystic neoplasm with low-grade epithelial atypia.

*Category 5* (pancreatobiliary neoplasm high risk/grade) includes features of an intraductal and/or cystic neoplasm with high-grade epithelial atypia. High-grade epithelial atypia is defined as a cell smaller than a duodenal enterocyte, showing a high nuclear/cytoplasmic ratio and abnormal chromatin, which can be hypochromatic or hyperchromatic, with or without a necrotic background.

*Category 6* (suspicious for malignancy) includes FNA specimens showing cytopathological features suggestive of malignancy that, however, are insufficient in either number or quality to make an unequivocal diagnosis of malignancy. The use of this category should be limited, and when used, the report should include the type of malignant tumor suspected. Criteria used to assign categories 6 or 7 are qualitative rather than quantitative. It is worth noting that scant cellular FNA smears showing rare cells with features suggestive of neuroendocrine neoplasm should be assigned to this category (category 6—suspicious for malignancy) rather than category 3 (atypical).

*Category 7* (malignant) is represented by FNA smears showing unequivocal cytopathological features of malignancy and includes both primary and metastatic malignancies.

### Cytological Features of Pancreatic Neuroendocrine Neoplasms and Differential Diagnoses

**Fig. 1:**
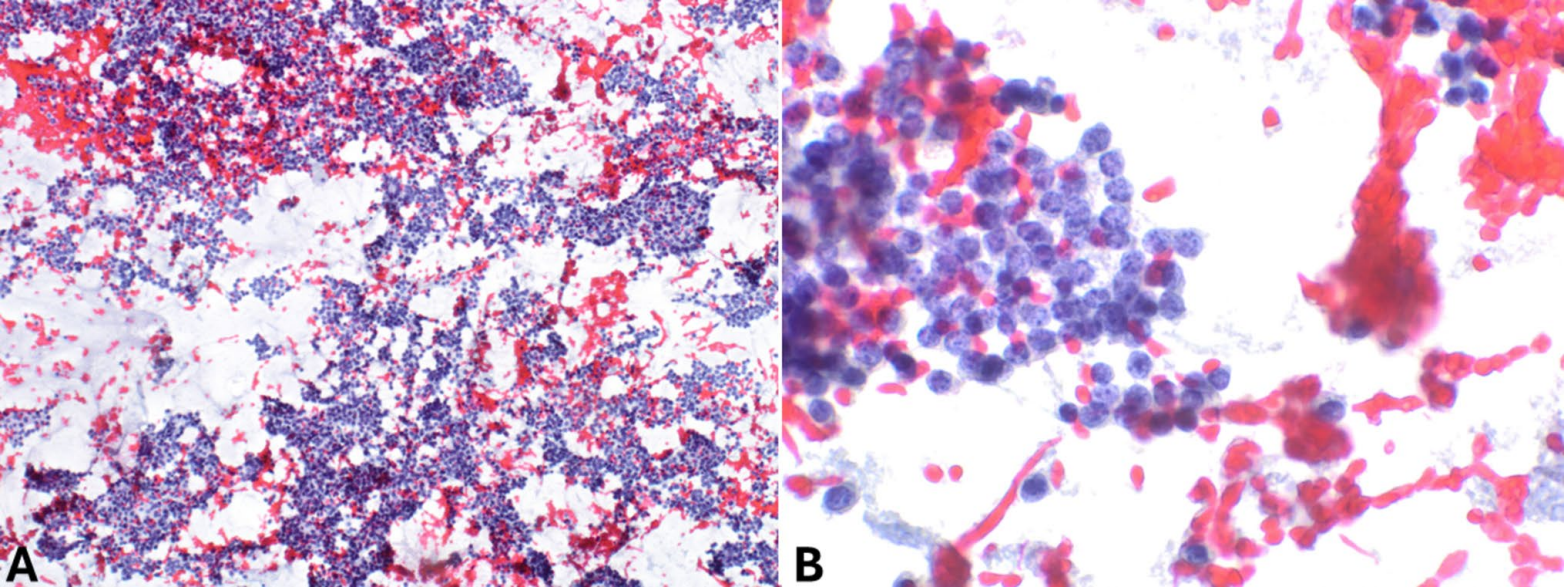
**A** Low power magnification view of pancreatic neuroendocrine tumor smear showing high cellularity in a hemorrhagic background (PAP stain). **B** At higher magnification, tumor cells appear uniform, small to medium in size with pale cytoplasm and eccentric round to oval nuclei with finely stippled and uniformly distributed chromatin (“salt and pepper” pattern)

**Fig. 2:**
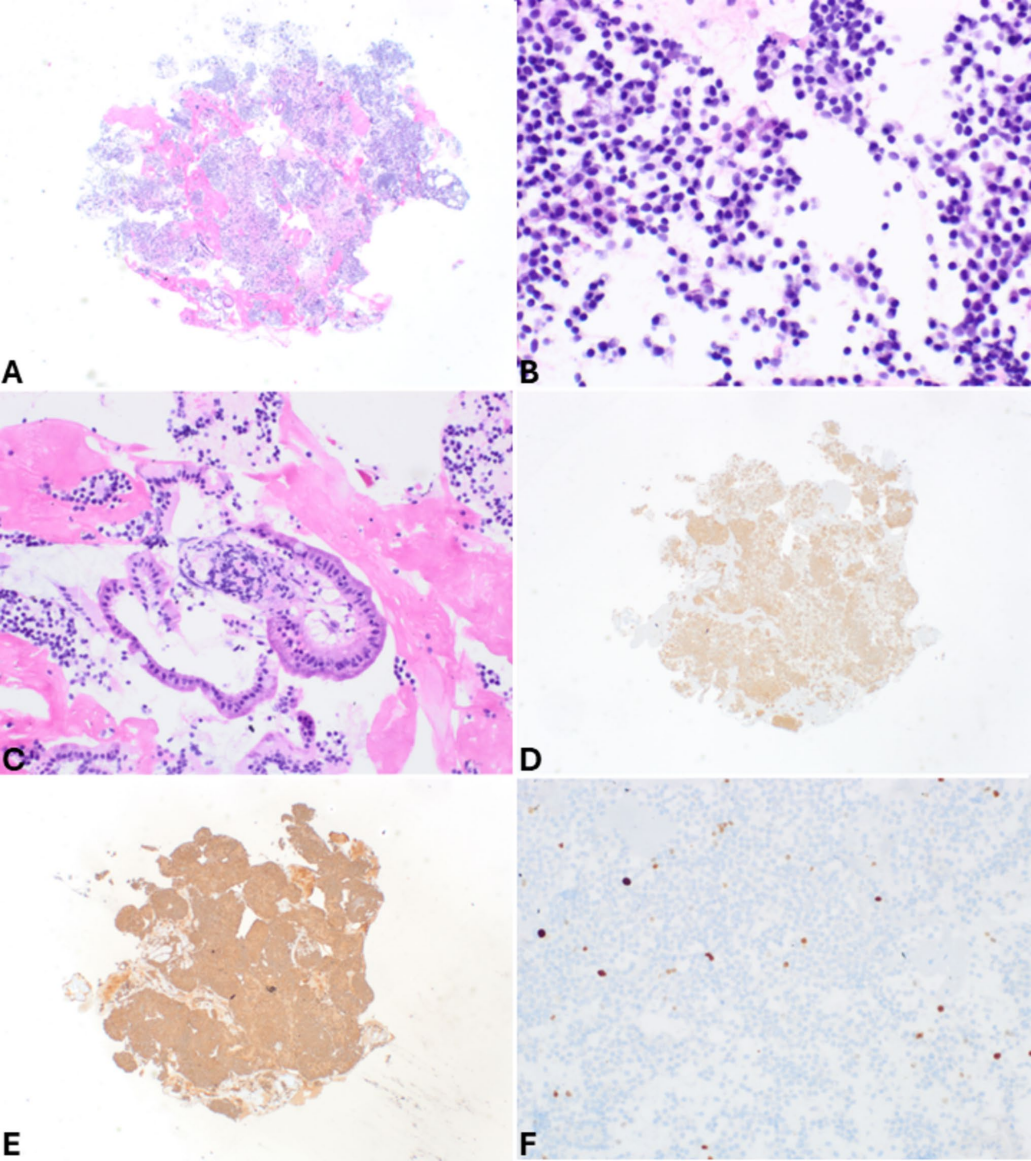
Cell block of the case shown in Fig. 1. At low power magnification (**A**), it appears highly cellular. At higher magnification (**B**), tumor cells are monomorph with round uniform nuclei eccentrically located, giving a plasmacytoid appearance. It is worth noting that contaminant normal gastric or duodenal (**C**) cells can be present in both FNA and FNB specimens, representing entrapped cells in the needle during the pass through the mucosa. General neuroendocrine markers including synaptophysin (**D**) and chromogranin A (**E**) are well expressed. The Ki67-proliferative index is low (**F**) conversely to what is observed in neuroendocrine carcinomas (see Fig. 3F)

EUS-FNA has a relatively high sensitivity for diagnosing panNENs, as nondiagnostic or false negative FNA reports are very rare and limited to small tumors [11]. In cystic lesions, biochemical tests on cystic fluids should be included in the diagnostic work-up, particularly the determination of carcinoembryonic antigen (CEA) and amylase levels, to aid in the differential diagnosis with intraductal papillary mucinous neoplasm (IPMN), mucinous cystic neoplasm, and cystic acinar cell carcinoma [12, 13]. FNA smears of pancreatic neuroendocrine tumors (panNETs) (Fig. 1) are usually highly or moderately cellular with uniform and monotonous cells forming loosely cohesive groups and rosette-like structures. Tumor cells are relatively small, occasionally medium-sized, with pale cytoplasm and indistinct cell borders. Nuclei are often eccentric (plasmacytoid appearance) and round to oval with finely stippled and uniformly distributed chromatin (“salt and pepper” pattern). Nucleoli are small and inconspicuous. The background can be hemorrhagic, and mitotic figures and necrotic cell debris are rarely observed [14]. The availability of a cell block enables the application of immunohistochemistry to confirm the neuroendocrine nature of the lesion (Fig. 2).

### Differential Diagnosis Between PanNETs and Mimicker Neoplasms

PanNETs need to be separated from several different neuroendocrine and non-neuroendocrine neoplasms, which may show similar cytological features. The distinction is of crucial clinical relevance since their management and prognosis are different. The most important differential diagnoses include pancreatic neuroendocrine carcinoma (panNEC), solid pseudopapillary neoplasm (SPN), and acinar cell carcinoma (ACC) (Table 2). 

**Table 2:** Cytological features of pancreatic neuroendocrine neoplasms and differential diagnoses

Cytological features	Entity				
	PanNET	PanNEC		SPN	ACC
		*Large cell*	*Small cell*		
Cellularity	High or moderate	High or moderate	High	High	High
Background	Can be hemorrhagic	Necrotic	Necrotic, crushed cells, chromatinic smearing	Can be hemorrhagic	Necrosis
Aggregation pattern	Loosely cohesive groups and rosette-like structures	Loose crowded fragments, occasionally three-dimensional, and/or dispersed single cells	Loose crowded fragments, occasionally three-dimensional, and/or dispersed single cells	Fragments with branching papillary arrangements composed of delicate fibrovascular cores and microadenoid structures	Three-dimensional fragments that show lobulated, trabecular, and small acinar architecture
Cytoplasm	Uniform and monotonous cells, small and medium in size; pale cytoplasm and indistinct cell borders	Large cells, with abundant eosinophilic cytoplasm	Small cells with minimal to imperceptible cytoplasm	Monotonous cuboidal neoplastic cells with granular or finely vacuolated cytoplasm with wispy borders	Eccentric, finely granular blue (Giemsa), aqua (Pap), or eosinophilic (H&E) granular cytoplasm with indistinct cytoplasmic membranes, which varies from abundant to very scant; minute clear vacuoles
Nuclei	Eccentric nuclei, round to oval Nucleoli are small and inconspicuous	Markedly enlarged nuclei, thick, relatively smooth nuclear membrane, prominent nucleoli	Angulated pleomorphic nuclei with molding scant nucleoli, mitotic figures and apoptotic bodies	Round or oval, eccentric nuclei with small nucleoli and nuclear grooves	Large nuclei, variable anisonucleosis, and prominent nucleoli
Chromatin pattern	“Salt and pepper”	Coarsely stippled chromatin, vesicular in Pap-stained smears	Hyperchromatic and coarsely stippled chromatin	With finely granular chromatin	Coarsely clumped chromatin

*ACC*, acinar cell carcinoma; *panNEC*, pancreatic neuroendocrine carcinoma; *panNET*, pancreatic neuroendocrine tumor; *SPN*, solid pseudopapillary neoplasm; *WHO*, World Health Organization

**Fig. 3:**
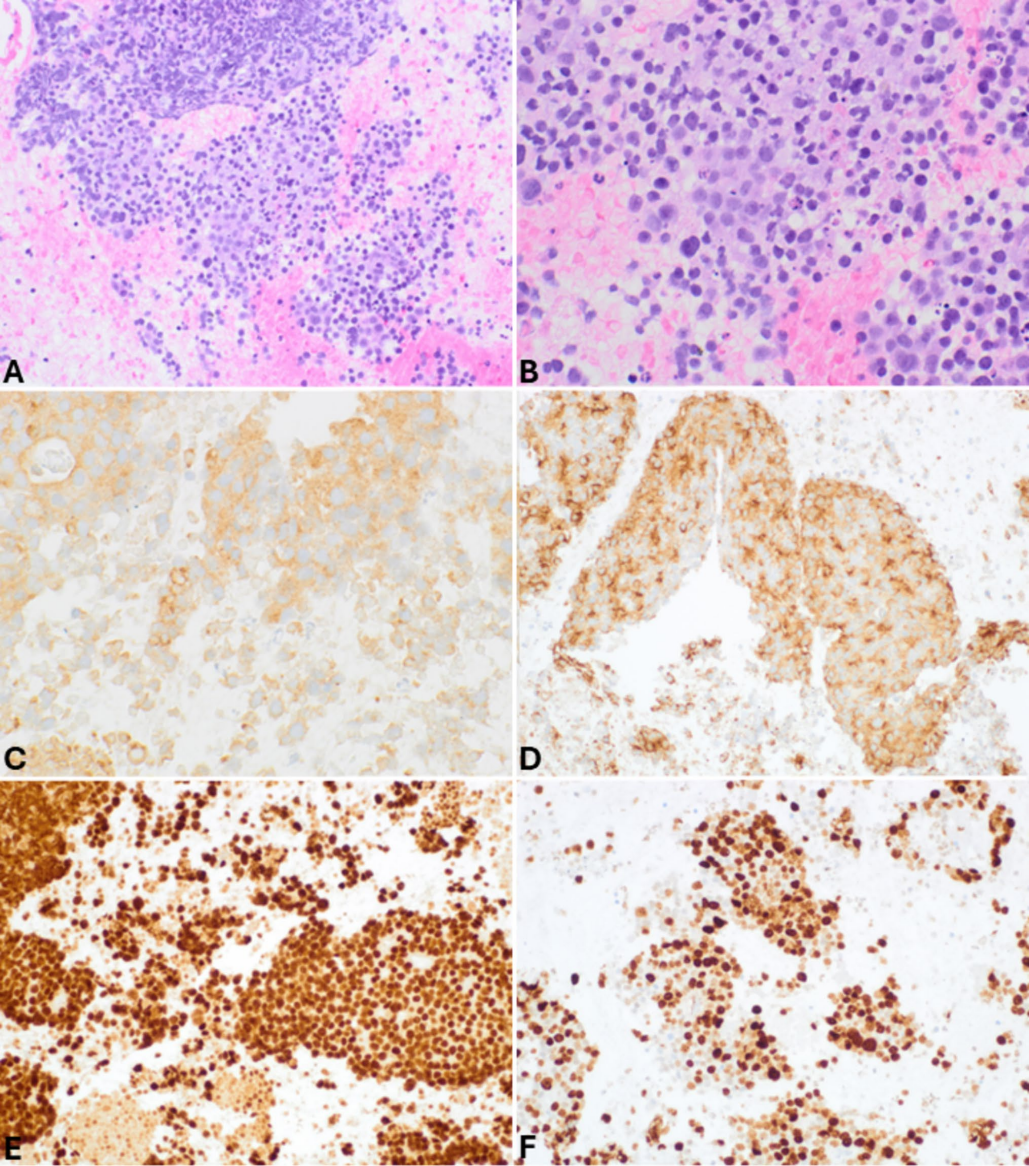
Low power magnification view (**A**) of large cell pancreatic neuroendocrine carcinoma showing high cellularity and necrosis (H&E stain). At higher magnification (**B**), tumor cells show abundant eosinophilic cytoplasm and large nuclei with coarsely stippled chromatin, sometimes nucleolated. Several apoptotic bodies are well evident. In this case, a cell block was performed, and immunohistochemistry demonstrated immunoreactivity for synaptophysin (**C**), chromogranin A (**D**), TTF1 (**E**), and high Ki67-proliferative index (**F**)

**Fig. 4:**
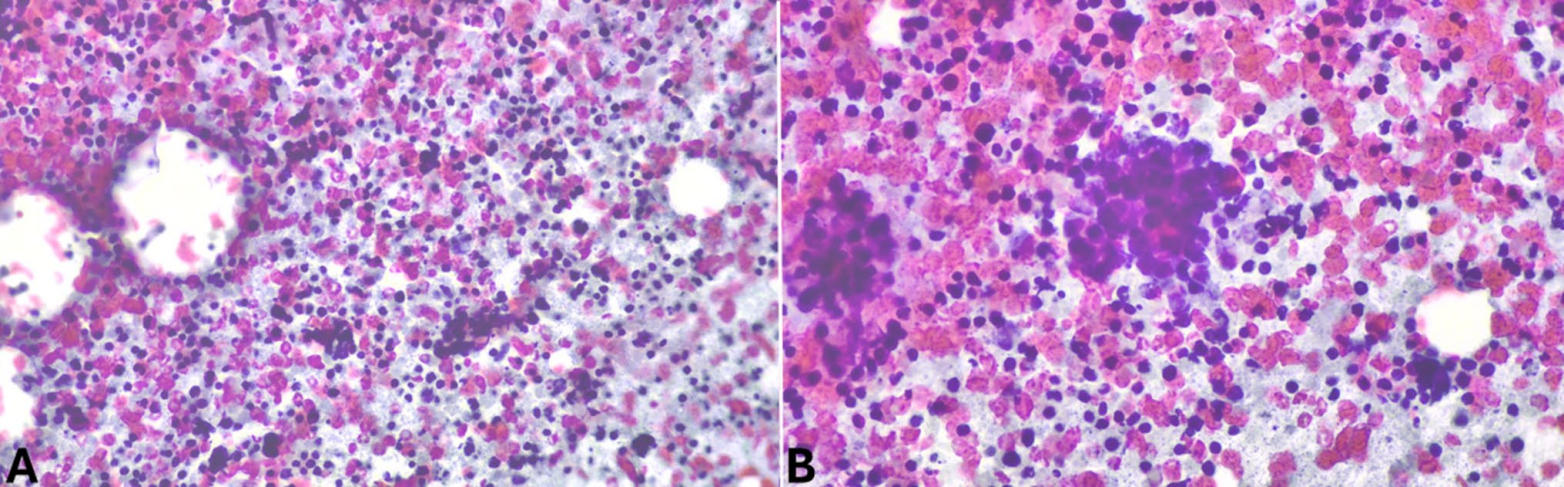
**A** Low power magnification view of highly cellular smear of small cell pancreatic neuroendocrine carcinoma with a dirty background and cells forming three-dimensional structures or dispersed as single cells. **B** At higher magnification, cells are markedly atypical, showing angulated pleomorphic hyperchromatic nuclei without nucleoli and scarce cytoplasm (Pap stain)

*Pancreatic neuroendocrine carcinomas (panNECs)* are classified into large and small cell subtypes, with the former being the most challenging to differentiate. In this context, grade 3 panNETs may represent a greater challenge for cytopathologists because they can exhibit moderate cell atypia. However, certain features support the diagnosis of panNETs, such as plasmacytoid morphology, smooth nuclear contours, and round nuclei. In contrast, large cell panNECs are composed of neoplastic cells showing abundant eosinophilic cytoplasm and markedly enlarged nuclei with coarsely stippled chromatin and prominent nucleoli. The nuclei in Pap-stained smears are vesicular and show a thickened, relatively smooth nuclear membrane (Fig. 3). Necrosis is frequently observed [15]. Key diagnostic cytopathological features of small cell panNEC include highly cellular smears composed of loose, occasionally three-dimensional, crowded tissue fragments, and/or dispersed single cells. Necrosis and crushed cells with chromatinic smearing are commonly observed. Additional features include angulated pleomorphic nuclei with molding, hyperchromatic and coarsely stippled chromatin, scant nucleoli, minimal to imperceptible cytoplasm, mitotic figures, and apoptotic bodies (Fig. 4) [10]. If a cell block is available, immunostaining for Ki67 is especially useful since the Ki67-proliferative index is extremely high in panNECs compared to panNETs [16, 17]. The aberrant expression of p53 and Rb, which reflects the related molecular alterations, can also serve as additional diagnostic tools [18].

**Fig. 5:**
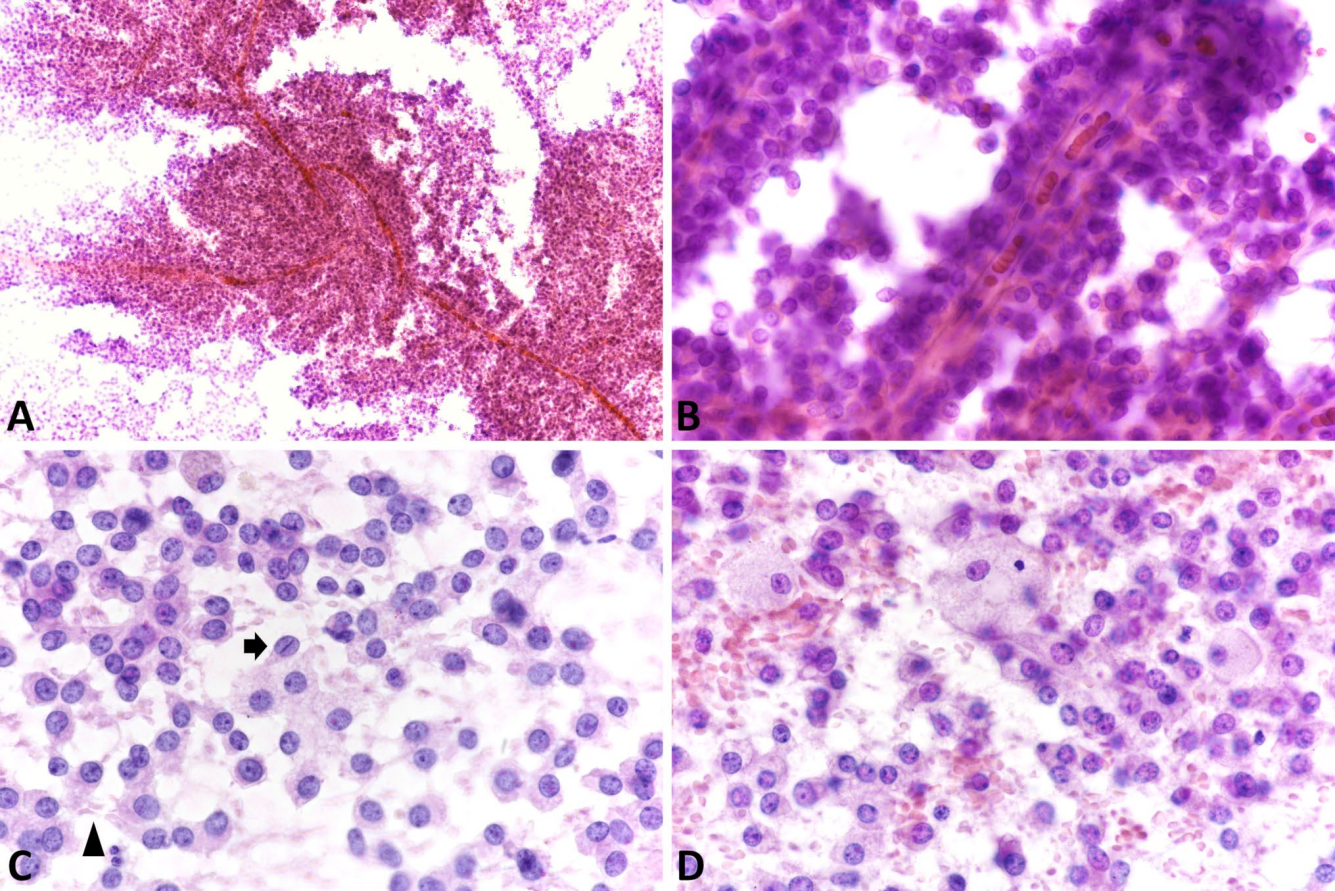
Low power magnification view of solid pseudopapillary neoplasm showing papillary structures of branching capillaries surrounded by discohesive neoplastic cells (**A**, Pap stain). At higher magnification (**B**), tumor cells appear small and monomorphic and show a tendency to detach from the papillae. They show slight nuclear atypia, nuclear grooves (arrow), and cytoplasm with a delicate elongation (some indicated by the arrowhead), the so-called cercariform cells (**C**). In addition to the monotonous cell population, another characteristic is the presence of a hemorrhagic background containing macrophages and/or giant cells (**D**) (reprinted from La Rosa S and Bongiovanni M “Pancreatic Solid Pseudopapillary Neoplasm: Key Pathologic and Genetic Features” (Arch Pathol Lab Med. 2020;144(7):829–837) with permission from Archives of Pathology & Laboratory Medicine. Copyright 2020. College of American Pathologists, reference 47)

*Solid pseudopapillary neoplasm (SPN)* usually presents hypercellular smears with characteristic branching papillary arrangements composed of delicate fibrovascular cores and monotonous cuboidal neoplastic cells. The nuclei are round or oval, eccentric with finely granular chromatin, small nucleoli, and frequent nuclear grooves. The cytoplasm is granular or finely vacuolated with wispy borders. Mitotic activity or significant atypia are rarely observed (Fig. 5). The presence of rosettes without papillary structures and the typical “salt and pepper” chromatin are lacking. The architectural features of SPN including pseudopapillae and solid discohesive sheets are more evident in cell block sections where useful immunohistochemical stains can be performed (see following sections) [19, 20].

**Fig. 6:**
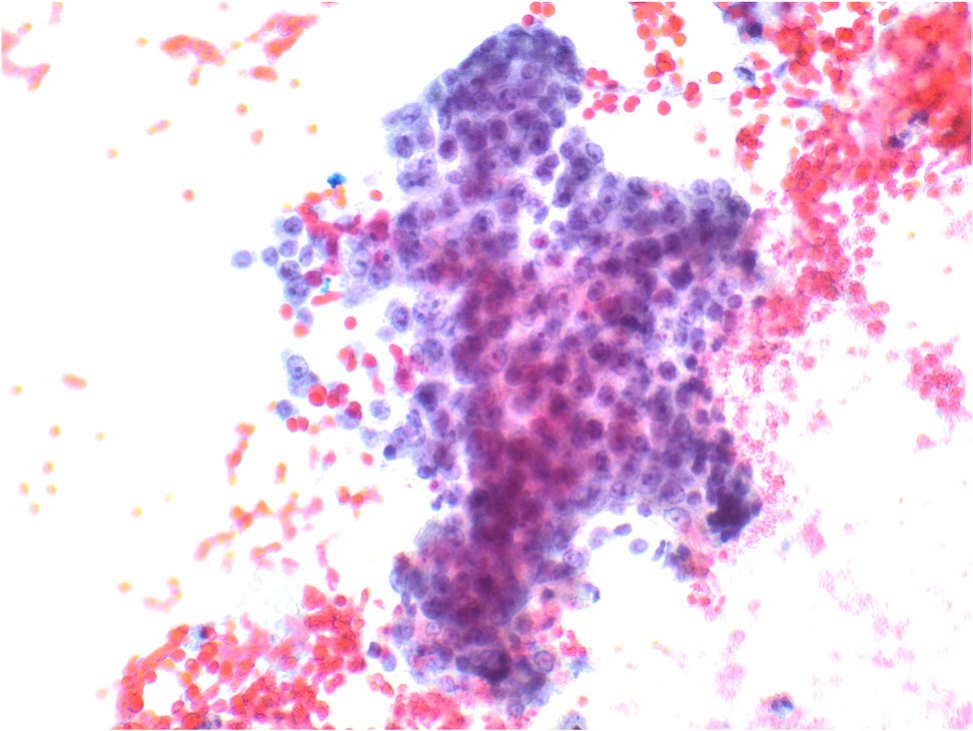
Pancreatic acinar cell carcinoma smear showing hypercellular sheet of neoplastic cells with large nuclei with coarsely clumped chromatin and prominent nucleoli and eccentric, finely granular aqua cytoplasm with indistinct cytoplasmic membranes (PAP stain)

**Fig. 7:**
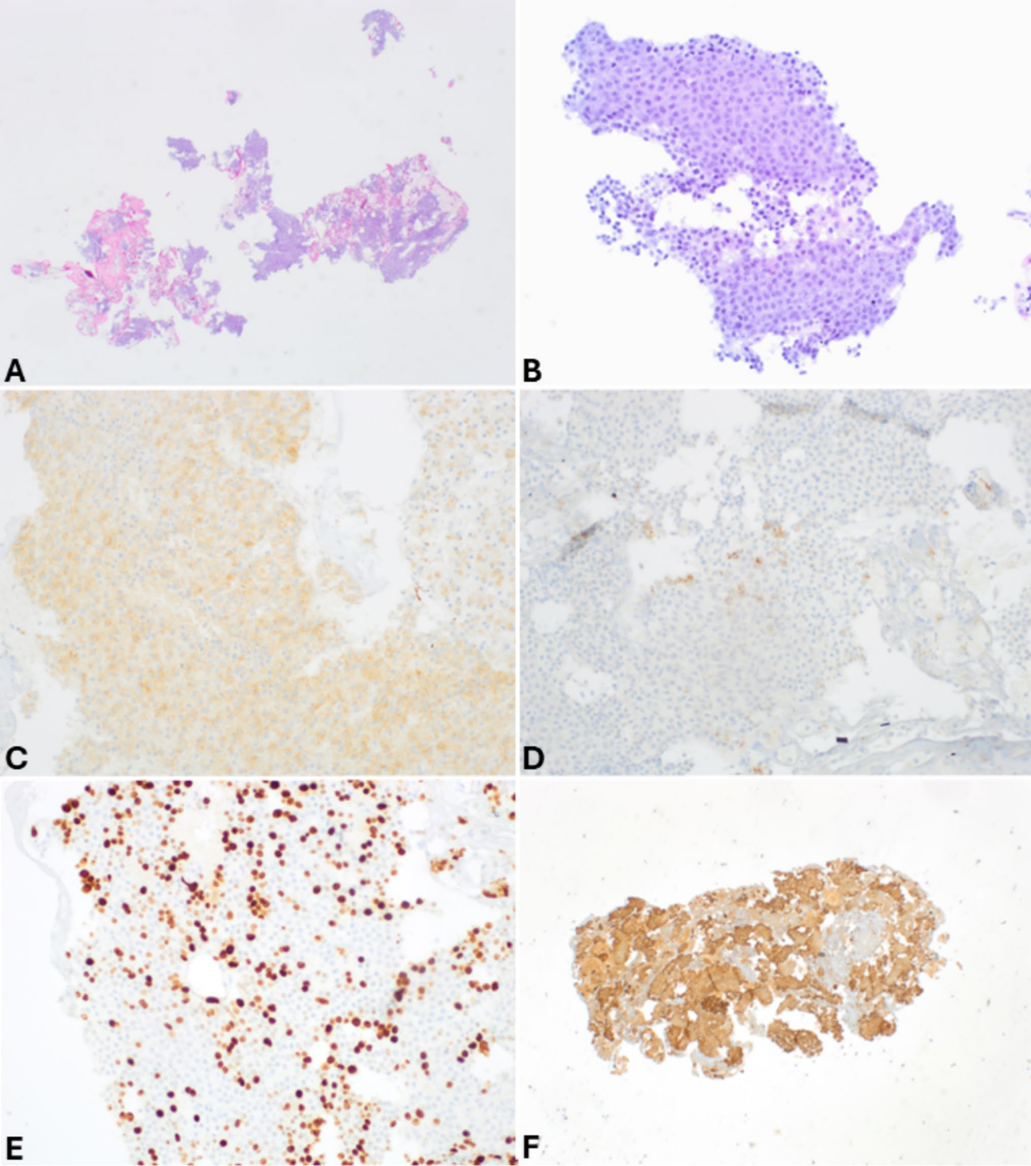
Cell block of the case shown in Fig. 6. At low power magnification (**A**), it appears highly cellular, and at higher magnification (**B**), tumor cells are large with abundant eosinophilic cytoplasm and large, clear, and nucleolated nuclei. In this case, synaptophysin is positive in several cells (**C**), while chromogranin A is expressed by scattered cells (**D**). It is worth noting that neuroendocrine markers are expressed in about 30% of acinar cell carcinomas; therefore, in the presence of acinar morphological features (image B) and a high Ki67-proliferative index (**E**), the search for acinar cell markers such as trypsin (**F**) and bcl10 is essential to avoid a wrong diagnosis

*Acinar cell carcinoma (ACC)* is a crucial differential diagnosis, particularly in advanced disease, due to substantial differences in tumor prognosis and therapeutic implications. Key cytopathological features of ACC (Fig. 6) include hypercellular smears composed of dense, three-dimensional fragments that show lobulated, trabecular, and small acinar architecture. The tumor cells are pyramidal and epithelioid with large nuclei, prominent nucleoli, and variable anisonucleosis. The cytoplasm is variable in size, eccentric, finely granular blue (Giemsa), aqua (Pap), or eosinophilic (H&E) granular. Minute clear vacuoles representing negative images of zymogen granules are seen on Giemsa-stained smears [21–23]. Some of these features (high cellular smears, marked anisonucleosis, and moderate to abundant eosinophilic cytoplasm) can also be observed in large cell NECs, which should be distinguished from ACC. Large cell NECs typically show abundant necrosis, loosely crowded tissue fragments and/or dispersed single cells with thickened and relatively smooth nuclear membranes, and markedly enlarged vesicular nuclei (on Pap-stained smears) with coarsely stippled chromatin and prominent nucleoli. Acinar cell differentiation can be confirmed by immunohistochemistry for BCL10 or trypsin, when a cell block is available (Fig. 7) [24].

Cytologists need to be aware that some rare primary neoplasms or pancreatic metastases can show neuroendocrine differentiation, such as pancreatic anaplastic carcinoma [25], pancreatic mixed acinar-neuroendocrine carcinoma [26, 27], paraganglioma [28], and metastases of neuroendocrine tumors located in other organs [29, 30]. In this context, the availability of a cell block is of fundamental importance to properly manage the immunohistochemical tests (see following sections) [20].

## Histological Features in FNB Specimens

### Neoplasms with Neuroendocrine-Like Features

The term “neuroendocrine-like features” is routinely used to indicate specific architectural and cytological aspects that characterize neuroendocrine neoplasms (NENs), and its recognition is the first step of the histological diagnosis [31]. Trabecular, insular, nested, or alveolar architectures represent the typical structures observed in NENs, although other rarer patterns of growth can be encountered, including glandular, paraganglioma-like, and solid structures. In addition to the architecture, tumor cells’ morphology is quite characteristic. These features also aid in distinguishing well-differentiated neuroendocrine tumors (NETs) from poorly differentiated neuroendocrine carcinomas (NECs).

**Fig. 8:**
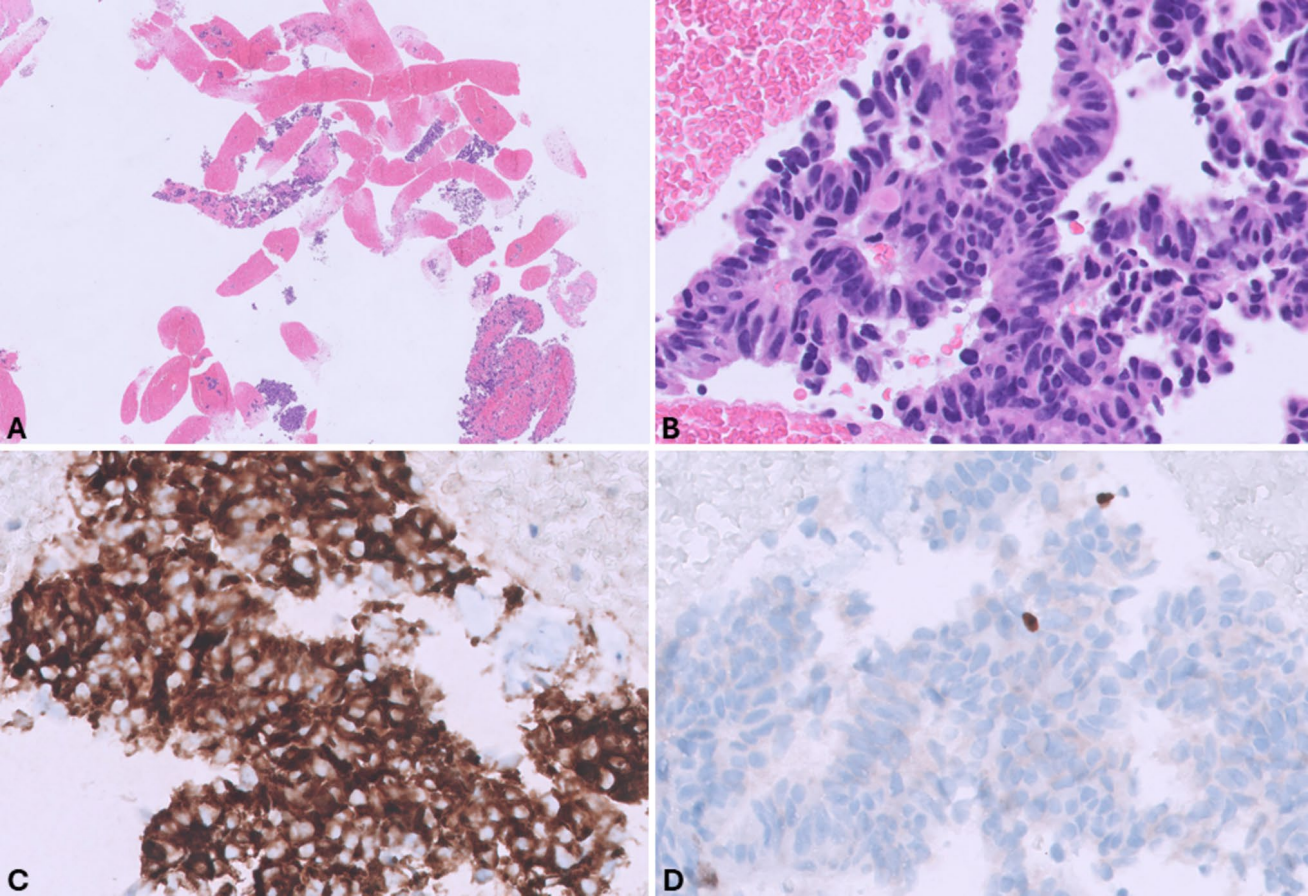
**A** Low power magnification view FNB of panNET. **B** At higher magnification, the tumor is composed of well-differentiated cells with uniform round nuclei without nucleoli and eosinophilic cytoplasm forming a trabecular/gyriform structures. **C** Tumor cells are strongly positive for chromogranin A, and the Ki67-proliferative index is low (courtesy of prof. Silvia Uccella, Humanitas University, Milan, Italy)

NETs are composed of rather monomorphic cells with abundant eosinophilic cytoplasm and round, monomorphic nuclei with coarsely stippled nuclear “salt and pepper” chromatin (Fig. 8). Mitoses are rare or absent. However, in some cases, tumor cells have an irregular size, increased nuclear/cytoplasmic ratio, prominent nucleoli, and mitoses. In general, these cases are associated with an increased Ki67-proliferative index (> 20%).

In contrast, NECs are composed of very atypical cells, with large cell or small cell morphology. Small cell NECs are composed of neoplastic elements not larger than three times a resting lymphocyte, with scant cytoplasm and hyperchromatic nuclei without nucleoli. In large cell NECs, tumor cells are larger than three resting lymphocytes, with vesicular nuclei showing prominent nucleoli and abundant eosinophilic cytoplasm. Necrosis and high mitotic activity are typically present in both subgroups.

Since “neuroendocrine-like features” can also be observed in non-neuroendocrine neoplasms, the immunohistochemical confirmation of neuroendocrine phenotype using general neuroendocrine markers (synaptophysin, chromogranin A, and INSM1) is required [31]. While in surgical specimens, both architectural and cytological features can be adequately examined, this evaluation can be more problematic when working with low-cellularity specimens. In this context, an accurate balance of architecture, cytology, and immunophenotype (Table 3) is essential for an accurate diagnosis.

**Table 3:** Immunohistochemical markers useful for the differential diagnoses of pancreatic neuroendocrine tumors in FNB specimens

Markers	PanNET	NEC	MtsNET	SPN	ACC	PDAC	PB	Paraganglioma	NHL	EWS	MM
Cytokeratin	+	+	+	±	+	+	+	-	-	-/+	-
Chrom A	+	±	+	-	±	-	±	+	-	-/+	-
Syn	+	+	+	+	±	-	±	+	-	-/+	-
INSM1	+	+	+	-	±	-	±	+	-	-	-
p53	- (± in G3)	+	- (+ in G3)	-	-/+	+	-/+	-	-	±	-
Rb	+	-	+	+	+	+	+	+	-	+	-
SSTR2A	+	-/+	+	-	-	-	-	+	-	-	-
CD99	-	-/+	-	+ (dot-like)	-	-	-	-	-	+	-
Nuclear β cat	-	-	-	+	-/+	-	+ (nest)	-	-	variable	-
LEF1	-	-	-	+	-	-	-	-	variable	-	-
BCL10	-	-	-	-	+	-	+	-	-	-	-
Trypsin	-	-	-	-	+	-	+	-	variable	-	-
EMA	-	-	-	-	-	-	+ (nest)	-	-	-	-
S100	+	-	-	-	-	-	+	+	-	-/+	+
GATA3	-	-	-	-	-	-	-	+	-	-	-
CD45	-	-	-	-	-	-	-	-	+	-	-
CEA	-	-	-	-	-	+	-	-	-	-	-
SOX10	-	-	-	-	-	-	-	-	-	-	+
Mart-1	-	-	-	-	-	-	-	-	-	-	+
HMB-45	-	-	-	-	-	-	-	-	-	-	+

*panNET*, pancreatic neuroendocrine tumor; *NEC*, neuroendocrine carcinoma; *MtsNET*, metastatic NET; *SPN*, solid pseudopapillary neoplasm; *ACC*, acinar cell carcinoma; *PDAC*, pancreatic ductal adenocarcinoma; *PB*, pancreatoblastoma; *NHL*, non-Hodgkin lymphoma; *EWS*, Ewing sarcoma; *MM*, malignant melanoma; *Chrom A*, chromogranin A; *Syn*, synaptophysin; *SSTR2A*, somatostatin receptor 2 A; *Sub P*, substance P; *VMAT1*, vesicular monoamine transporter 1; *, ileal serotonin-producing NET

It is worth noting that there are at least five challenges in the diagnosis of panNENs in FNB:

I) Grading panNETs
II) Distinguishing panNETs from panNECs (either of small or large cell type)
III) Distinguishing panNETs from non-neuroendocrine mimickers
IV) Distinguishing small cell panNECs from non-neuroendocrine mimickers
V) Distinguishing large cell panNECs from non-neuroendocrine mimickers

### I) Grading panNETs

Tumor grading is based on both the mitotic and the Ki67-proliferative indices. The mitotic index is determined by counting the number of mitoses in a 2-mm^2^ area. However, this may be difficult to establish in small specimens and even impossible in cytological smears. For this reason, the mitotic index is generally not used as a parameter for tumor grading evaluation in FNA/FNB material. In contrast, establishing the Ki67-proliferative index is possible since it is determined by evaluating at least 500 tumor cells, which are typically present in both cytology and FNB material. However, it is worth noting that Ki67 expression in NENs is heterogeneous, requiring identification of hot spot areas for accurate evaluation [32]. For this reason, the best and most accurate evaluation of the Ki67 index can only be performed on surgical specimens and, possibly, using multiple blocks. Despite this limitation, determining Ki67 in limited cellularity samples remains of crucial importance, considering the impact of grading on the surgical management of small panNETs. For this reason, several studies have analyzed the performance of the Ki67 index in the determination of tumor grade in both FNA and FNB specimens by comparing it with the corresponding surgically resected tumors. Although several studies demonstrated good performance, others have found that they are not adequate for accurate grading [33–39]. The concordance between FNA/FNB and the corresponding surgical specimens ranged from 6 to 59%, with some authors demonstrating high [38] or moderate [39] concordance. In particular, it has been reported that the concordance depends on tumor grade. Indeed, the Ki67 index between FNA/FNB and surgical specimens was generally concordant for G3 panNETs, while discordant in 25% of G1 or G2 panNETs, suggesting a possible undergrading of FNB specimens in these categories [39]. In light of these observations, it clearly appears that the Ki67 index evaluation in FNB works well for high-grade panNETs, while for G1 and G2 tumors it should be considered with caution since it may be undergraded and clinicians should be aware of this issue.

### II) Distinguishing Between panNETs and panNECs (Either of Small or Large Cell Type)

**Fig. 9:**
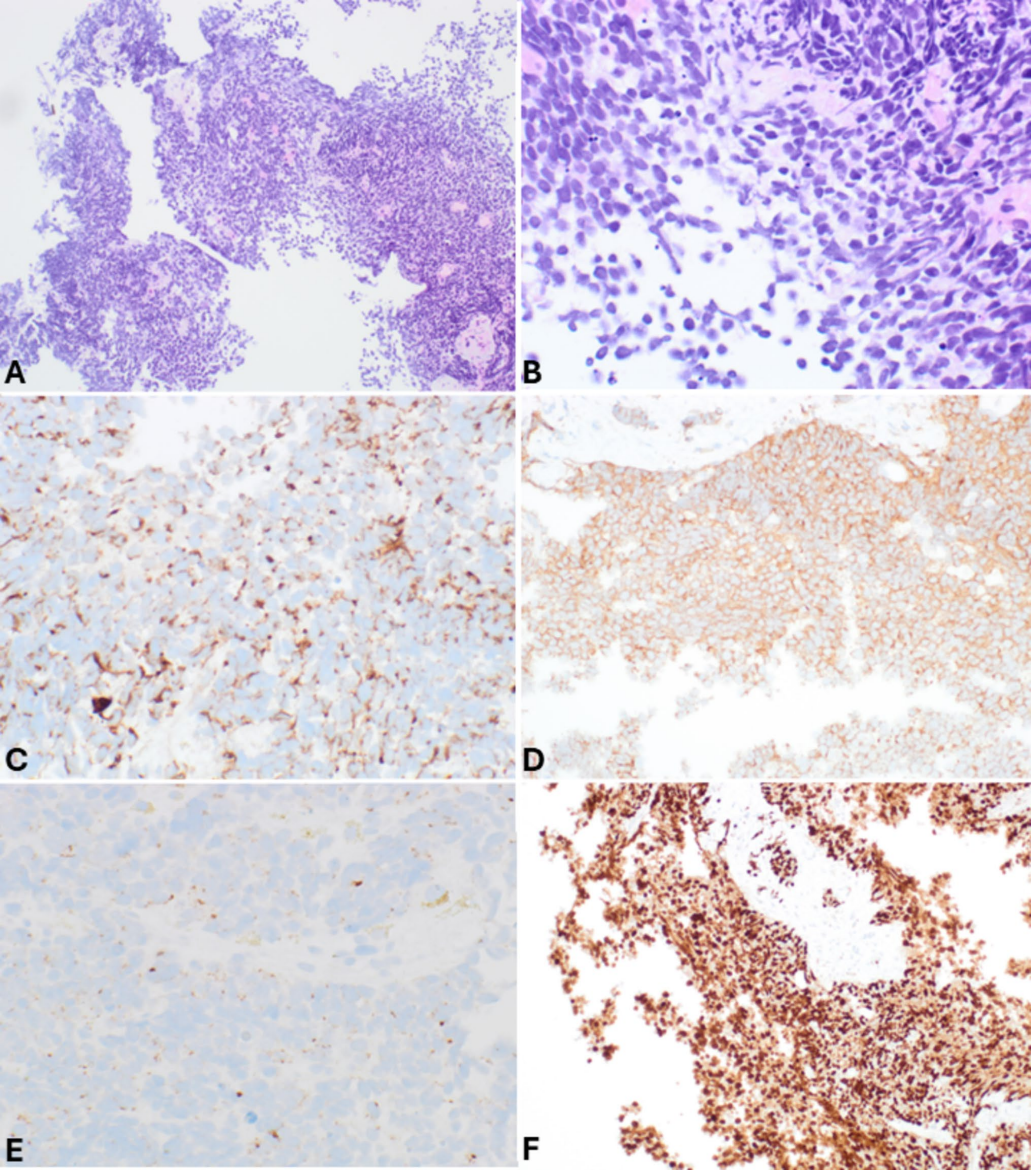
FNB specimen of small cell pancreatic neuroendocrine carcinoma. At low power magnification (**A**), the cancer appears highly cellular, and nuclear molding is well evident. At higher magnification (**B**), tumor cells are small and show hyperchromic nuclei without nucleoli and very scant cytoplasm. The epithelial nature of the neoplasm is confirmed by cytokeratin expression (**C**) that in small cell NEC can show a peculiar perinuclear dot-like expression. The neuroendocrine phenotype is demonstrated by synaptophysin immunoreactivity (**D**) and chromogranin A positivity (**E**), which is typically present in scattered cells with a perinuclear pattern corresponding to the low number of immature secretory granules mainly localized in the perinuclear Golgi area. Ki67-proliferative index is very high (**F**)

**Fig. 10:**
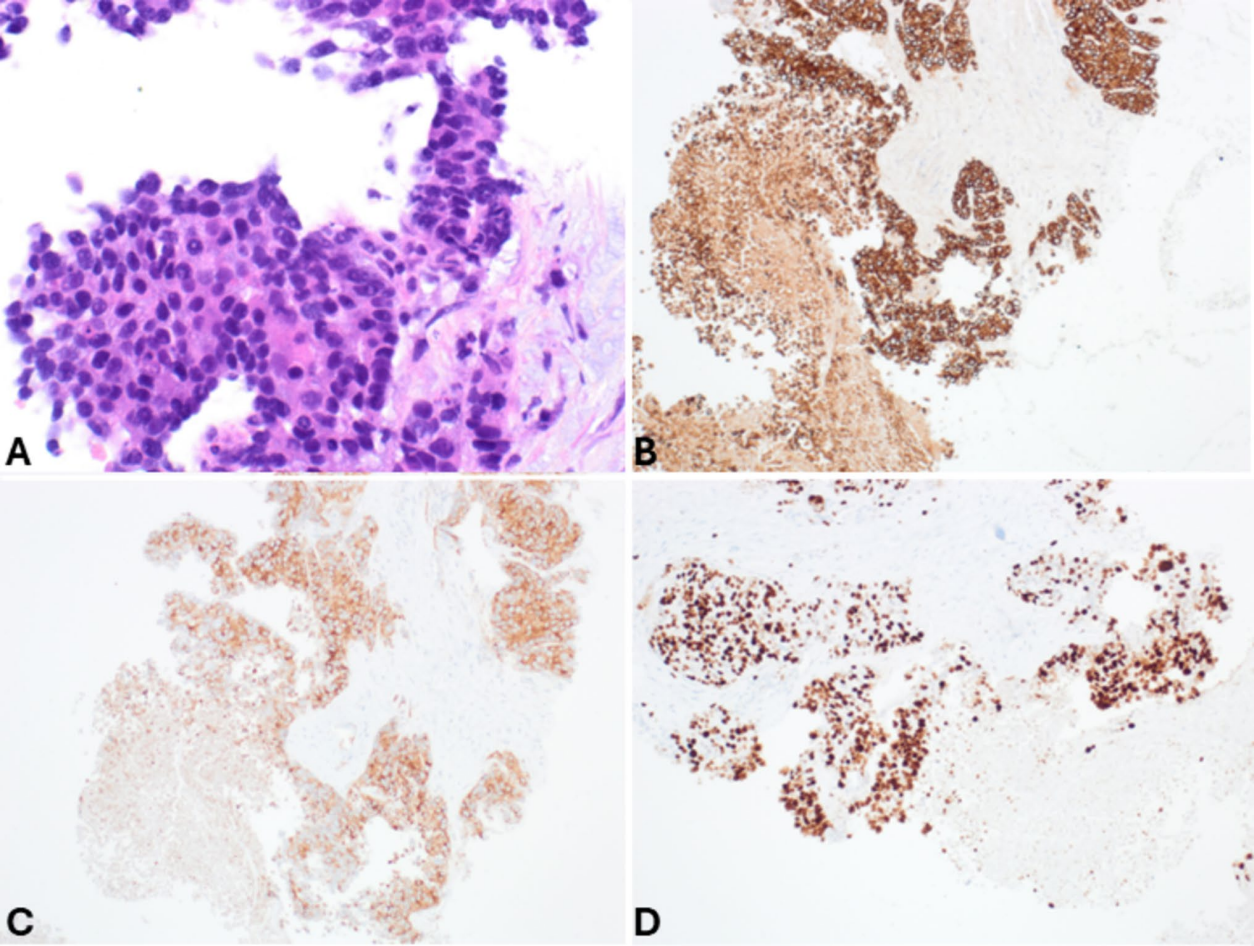
FNB specimen of pancreatic large cell neuroendocrine carcinoma. Tumor cells form a solid sheet, and, as frequently observed in this type of specimens, the large and vesicular nuclei with prominent nucleoli are not well evident due to crushing artifacts (**A**). However, tumor cells are markedly atypical with abundant eosinophilic cytoplasm. Some mitoses and apoptotic bodies can be observed. Tumor cells are strongly positive for cytokeratin (**B**) and general neuroendocrine markers including synaptophysin (**C**) and chromogranin A. The Ki67-proliferative index is high (**D**)

The distinction between panNETs and small cell panNECs is generally easy due to the different morphology of the tumor cells (Fig. 9), as is the distinction between large cell and small cell panNECs. On the contrary, in FNB specimens, the differential diagnosis between panNETs and large cell panNECs (Fig. 10) can be challenging and needs integration with immunohistochemical features and, sometimes, with the molecular profile. While general neuroendocrine markers are expressed in both categories, the aberrant expression of p53 and Rb can help. Indeed, since panNETs generally do not harbor mutations in both *TP53* and *RB1* genes, they show a “wild type” immunohistochemical expression of p53 and a strong Rb nuclear immunoreactivity. In contrast, mutations in these genes, frequently observed in panNECs, are associated with the lack of Rb positivity and either strong and diffuse expression or total negativity for p53 protein [18, 40, 41]. In addition, the lack of either DAXX or ATRX expression is a peculiar feature of panNETs, so it can support the diagnosis, although it is observed in only 30% of cases [18, 42]. The Ki67-proliferative index > 20% is not useful in this context because it can also be observed in G3 panNETs, although rarely it exceeds 55% as observed in panNECs [40]. However, Ki67 immunohistochemistry should always be performed in the work-up of NENs because it gives important information on the proliferative status of the neoplasm. It appears particularly useful when working on biopsy specimens with crush artifacts, which may simulate small cell NECs. In this context, an apparently “small cell morphology” associated with a low Ki67 immunolabelling should be considered very carefully and should avoid making the diagnosis of NEC. An additional finding that can be considered in this context is the lack of expression of the somatostatin receptor 2 A (SSTR2A) that can be observed in a subgroup of large cell panNECs, while it is strongly expressed at the cell membrane level in almost all panNETs, except for insulinomas [43, 44]. Reduced expression of chromogranin A can be observed in large cell panNECs [45], although it may be difficult to quantify in FNB specimens.

Since several G3 panNETs and panNECs are already metastatic at the time of initial diagnosis and not surgically resectable, the search for theragnostic biomarkers in small biopsy specimens is now relevant, and pathologists should be increasingly aware of this clinical need. In this context, immunotherapy is a useful and emerging therapeutic approach that requires careful selection of patients who can respond positively to the therapy. The integration of PD-L1 and mismatch repair protein (MMR) immunohistochemistry can be useful for identifying patients who would benefit from treatment with immune checkpoint inhibitors and can be performed in specific clinical contexts [46].

The progression of metastasized panNETs under systemic therapy, characterized by enlargement of metastases and/or an increased number of tumor lesions associated with deterioration in clinical condition, increased transaminases and circulating tumor markers, has been observed [47, 48]. This clinical progression is associated with an increase in the Ki67-proliferative index and the development of NEC-like features, including *TP53* and *RB* mutations. However, not all typical features of NEC are observed, and the lack of DAXX/ATRX expression or the retained menin immunohistochemical positivity is an important tool to identify these NEC-like panNETs and to distinguish them from pure NECs. Although this phenomenon is not well understood, it should be considered when biopsies of metastases showing NEC-like features are obtained from patients with a history of panNET [47–49].

### III) Distinguishing Between panNETs and Non-neuroendocrine Mimickers

Well-differentiated neuroendocrine-like features can be observed in several non-neuroendocrine neoplasms, which represent insidious mimickers that need to be identified because of their different clinical management and prognosis.

**Fig. 11:**
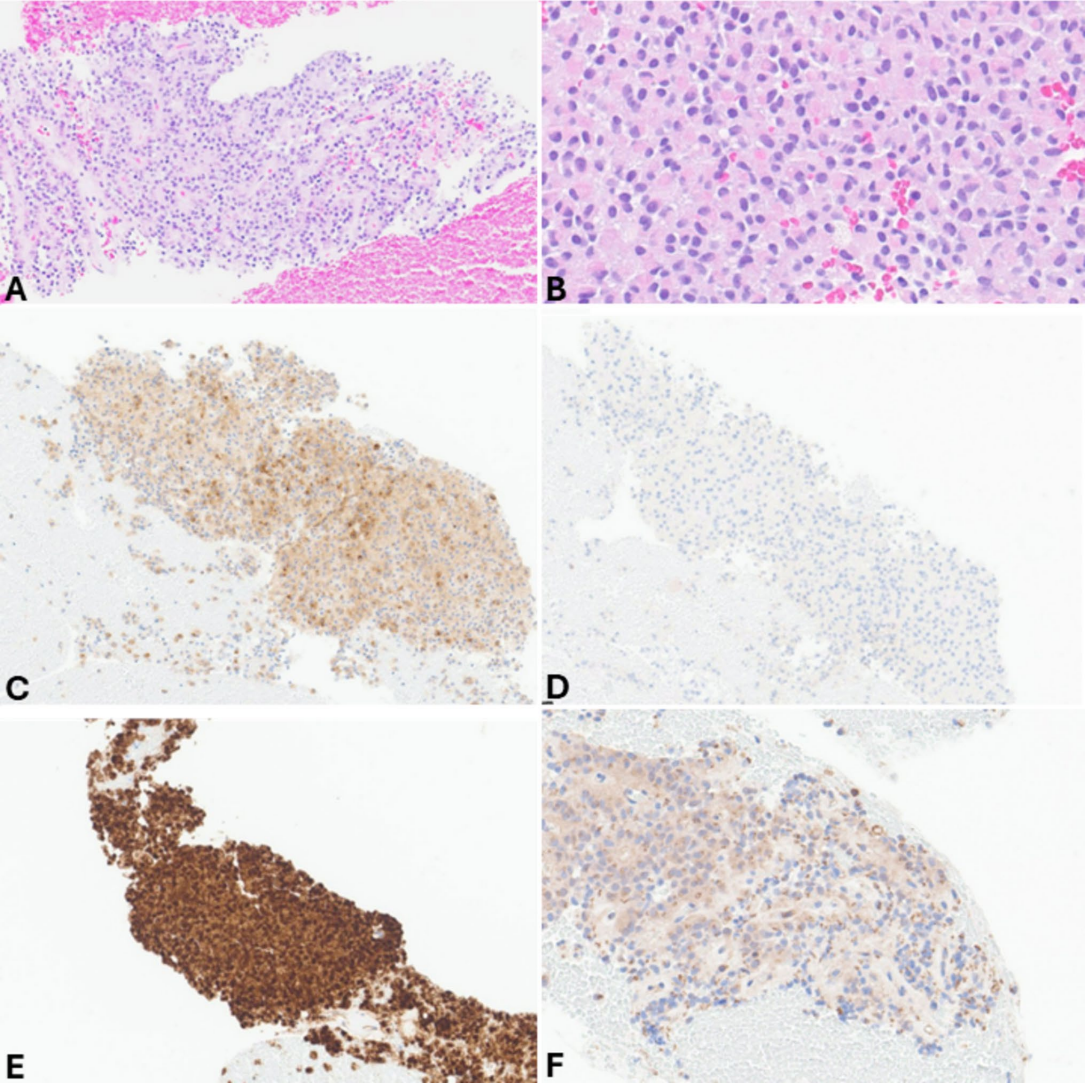
FNB specimen of solid pseudopapillary neoplasm showing solid growth with a tendency to lose cell cohesion and to form pseudopapillae, which are well evident in the left part of the image (**A**). At higher magnification, tumor cells show eosinophilic cytoplasm sometimes presenting fine vacuolization and uniform nuclei without nucleoli (**B**). Tumor cells are diffusely positive for synaptophysin (**C**) but lack chromogranin A expression (**D**). Nuclear expression of β-catenin (**E**) and the paranuclear dot-like immunoreactivity for CD99 (**F**) are specific diagnostic markers (courtesy of Prof. Christine Sempoux, University of Lausanne, Switzerland)

*Solid pseudopapillary neoplasms(SPN)* may represent a challenge since they are composed of monomorphic cells with round and regular nuclei, sometimes indented, and with finely dispersed chromatin without a prominent nucleolus and eosinophilic cytoplasm, which is sometimes vacuolated (Fig. 11). The characteristic pseudopapillae may sometimes be difficult to identify in FNB specimens due to tissue compression. The presence of hemorrhage, cholesterol crystals surrounded by foreign body giant cells, and intracytoplasmic small PASD-positive hyaline globules in tumor cells are additional characteristic features and of diagnostic help when present [50, 51]. The selection of the most appropriate immunohistochemical panel (Fig. 11) is essential considering the partially overlapping immunophenotype between panNET and SPN including positivity for synaptophysin and progesterone receptor. SPN shows a complex immunophenotype (i.e., cyclin D1, CD10, CD56, CD200, and SOX11 expression), but the expression of β-catenin [52], lymphoid enhancer-binding factor 1 (LEF1) [53], and CD99 (with a characteristic paranuclear dot-like expression) [54] is specific and particularly useful for distinguishing SPNs from panNETs. Chromogranin A expression is also useful since it is positive in panNETs and negative in SPNs. Consequently, when looking at a slide where morphology may open the differential diagnosis between panNETs and SPNs and the material is not enough for an extensive immunohistochemical investigation, the immunohistochemical study should be restricted to the expected positive (β-catenin and either CD99 or LEF1) and negative (chromogranin A) markers, avoiding the use of overlapping markers like synaptophysin, progesterone receptor, or non-specific markers such as vimentin, claudin-5, claudin-7, CD56, cyclin D1, and CD200 [55–58]. Recently, ABCD1 expression has been demonstrated to be a new sensitive and specific marker of SPNs, potentially useful in the differential diagnosis with other pancreatic neoplasms, but its use has not yet been incorporated into routine diagnostic work-up [59]. The aberrant expression of E-cadherin in SPNs can represent an additional interesting and potentially useful marker; two distinct patterns of immunoreactivity can be observed depending on the antibody used and include a nuclear E-cadherin positivity when using the antibody directed against the cytoplasmic domain and E-cadherin negativity when using the antibody directed against the extracellular domain [60].

*Acinar cell carcinoma (ACC)* is an aggressive pancreatic cancer that may mimic panNETs from which it must be distinguished because of its different clinical management and poor prognosis [61]. ACCs share some morphological features with panNETs such as trabecular or solid growth. More importantly, about 30% of ACCs focally express neuroendocrine markers, which in some cases, may be diffusely positive, like true neuroendocrine neoplasms [62]. Similar to panNETs, ACCs also show high cellularity, absence of fibrous stroma, and neoplastic cells with moderate amounts of granular eosinophilic cytoplasm and generally uniform nuclei. However, the presence of necrosis, high mitotic count, and prominent nucleoli in tumor cells should alert the pathologist to the possible diagnosis of ACC when looking at a tumor thought to be a panNET [61]. The search for additional acinar cell-specific morphological features such as acinar structures sometimes with minute lumina resembling normal acini can help in the diagnostic work-up. It is worth noting that uncommon variants including oncocytic, spindle, clear, and pleomorphic cell types have been described in ACCs [61, 62], and they are discussed in the following paragraphs since they represent a specific diagnostic challenge. Immunohistochemistry is essential for the differential diagnosis between panNETs and ACCs. As a rule, every time an apparent neuroendocrine neoplasm shows one of the morphological features described above (necrosis, high mitotic count, and prominent nucleoli), immunohistochemistry should not be restricted to neuroendocrine markers that can be frequently expressed in ACCs but must also include acinar cell markers like trypsin and BCL10. It is worth noting that BCL10 and trypsin can show variable expression in ACCs, so both should be employed to confirm or exclude the diagnosis of ACC [61].

**Fig. 12:**
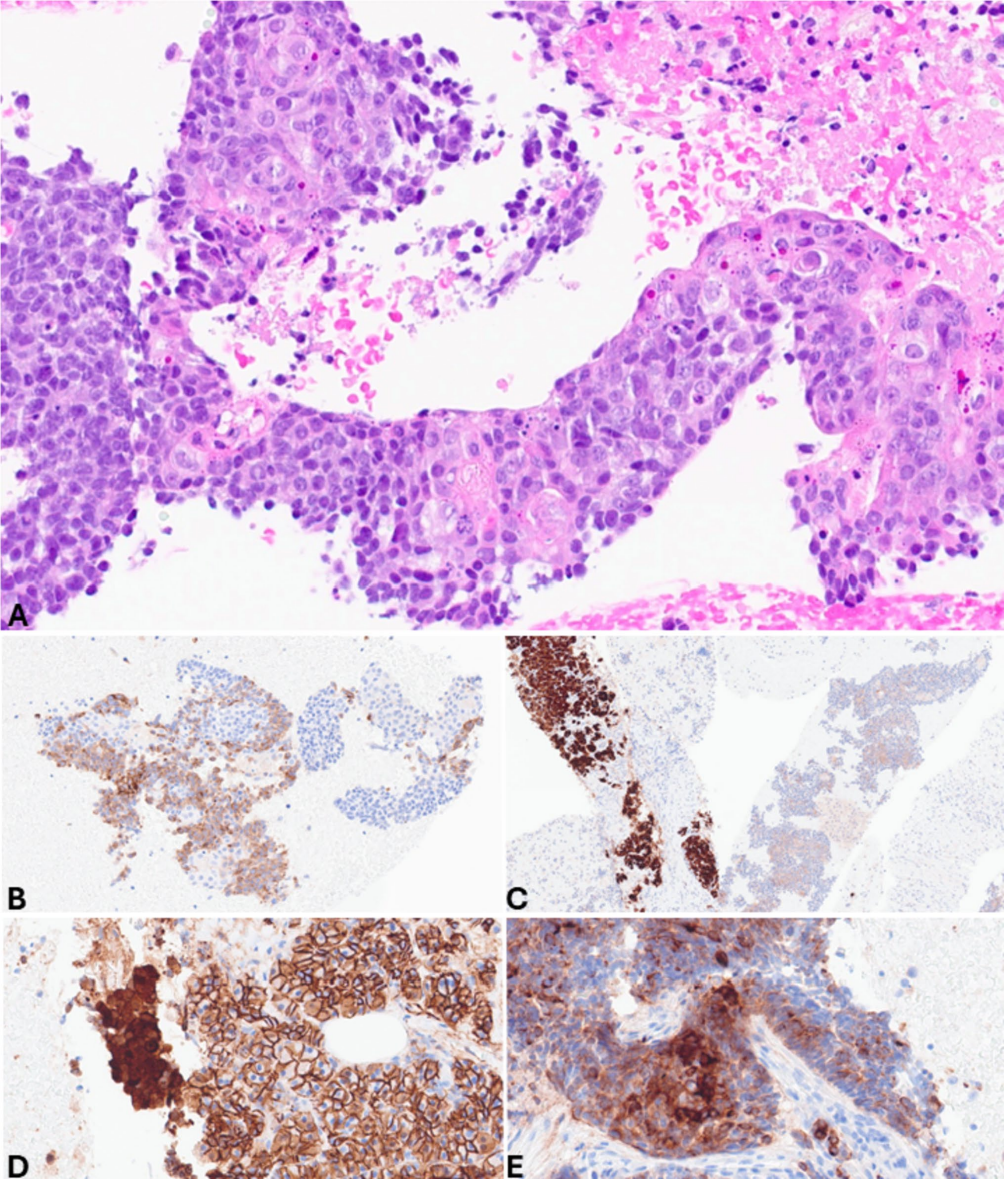
FNB specimen of pancreatoblastoma characterized by proliferation of atypical cells with abundant eosinophilic cytoplasm and vesicular nuclei with visible nucleoli associated with the presence of squamoid nests (**A**). In the upper right corner of the image, necrosis is well evident. Most tumor cells are positive for synaptophysin (**B**) and weakly for BCL10 (**C**, note the strong immunoreactivity in the normal pancreatic acini). Squamoid nests show nuclear immunoreactivity for β-catenin (**D**) and cytoplasmic immunostaining for EMA (**E**) (courtesy of Prof. Christine Sempoux, University of Lausanne, Switzerland)

The same diagnostic approach can be used for the differential diagnosis between panNETs and *pancreatoblastomas(PB)*, a complex pancreatic neoplasm showing a predominantly acinar differentiation. The distinction of PBs from ACCs lies in the presence of squamoid nests immunoreactive for β-catenin and EMA [63] (Fig. 12).

A particularly difficult task is the distinction of panNETs from *pancreatic metastases of NETs located elsewhere* [64, 65]. In this context, metastases from ileal NETs are a challenge since pancreatic serotonin-producing NETs do exist and represent 1.4% to 4% of panNETs [66–68]. Clinical history is essential to raise the suspicion of metastasis because the morphology of primary and metastatic serotonin-producing NETs is very similar, also including the presence of fibrous stroma [69]. However, the lack of substance P and VMAT1 expression and immunoreactivity for PDX1 and PAX6 in serotonin-producing panNETs may help in the differential diagnosis [67, 70]. In addition, unsupervised RNAseq-based clustering demonstrated that serotonin-producing panNETs were closer to nonfunctioning panNETs rather than ileal serotonin-producing EC-cell NETs [70]. For this reason, in selected cases, an integrated molecular analysis may be useful to get the final correct diagnosis. The expression of transcription factors CDX2, TTF1, and PAX8 can aid the differential diagnosis between panNETs and metastases from other NETs. CDX2 can be positive in about 15% of panNETs, but generally, its expression is less intense and patchy than that observed in intestinal EC-cell tumors [18]. TTF1 is negative in panNETs while it is positive in medullary thyroid carcinoma and in about 40% of lung NETs/carcinoids (most peripherally located). It is important to recall that lung NETs/carcinoids can be positive for calcitonin, so the search for CEA expression (only positive in medullary thyroid carcinoma) is essential in TTF1 metastatic NETs [18, 45]. PAX8 immunohistochemistry should be used with caution since different results can be obtained depending on which antibody is used. Polyclonal anti-PAX8 antibodies cross-react with other members of the PAX family (PAX3, PAX5, and PAX6) and can give positive staining in panNETs, likely due to cross reactivity with PAX6. On the contrary, monoclonal anti-PAX8 antibodies do not stain panNETs, and consequently, they should be used to distinguish between panNETs and metastatic NETs, such as those originating from the thymus [18].

**Fig. 13:**
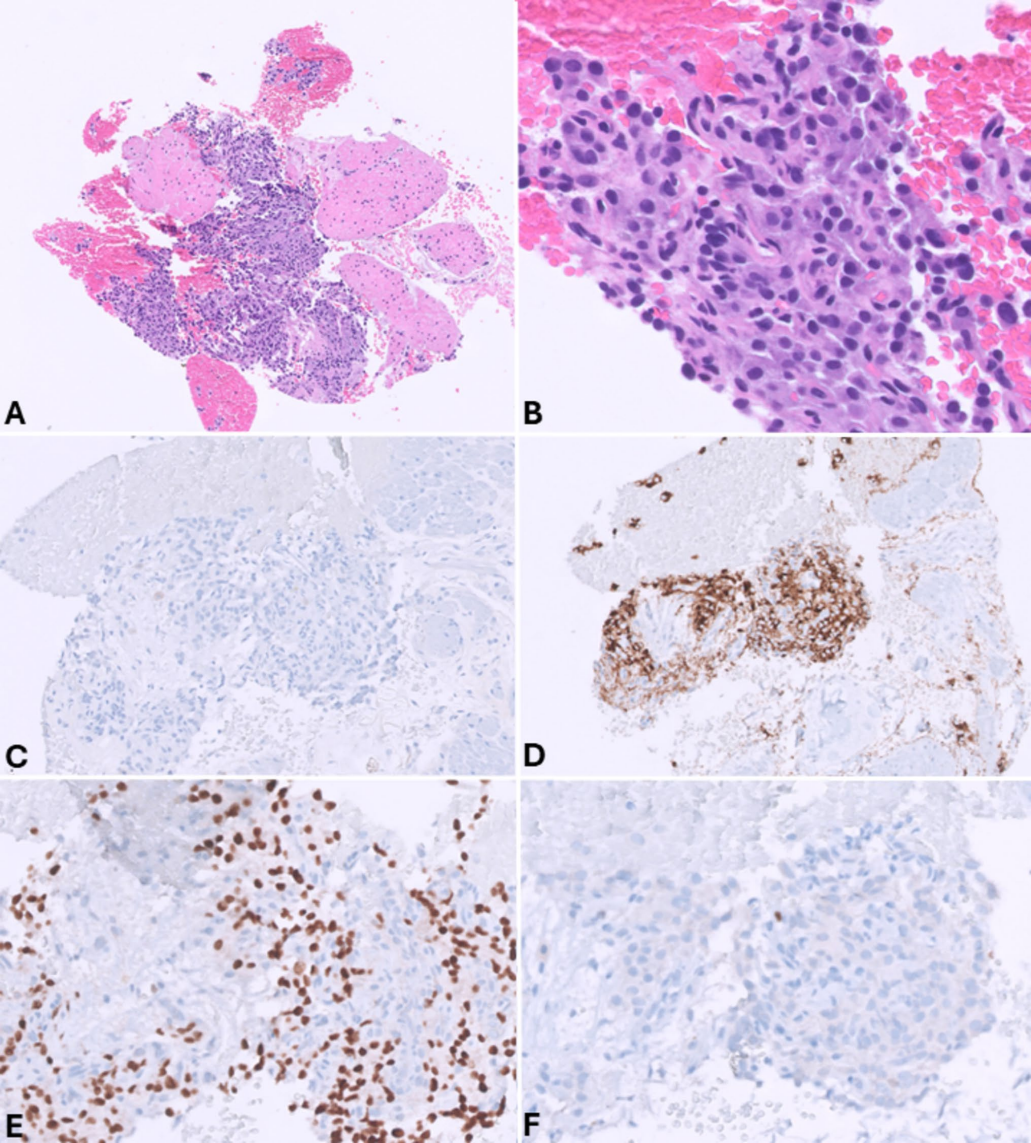
Low power magnification of FNB specimen of pancreatic paraganglioma (**A**). At higher magnification, cells are well differentiated with monomorphic round nuclei without nucleoli and abundant eosinophilic cytoplasm resembling cells of a pancreatic neuroendocrine tumor (**B**). However, tumor cells are negative for cytokeratin CAM 5.2 (**C**). Chromogranin A (**D**) and GATA3 (**E**) are diffusely positive. The Ki67-proliferative index is very low (**F**) (courtesy of prof. Silvia Uccella, Humanitas University, Milan, Italy)

*Extra-adrenal paraganglioma* is a non-epithelial neuroendocrine tumor that may rarely arise in the pancreas or in peripancreatic tissue. The organoid nested architecture and cells showing regular nuclei and basophilic cytoplasm (Fig. 13) may simulate a panNET, with particular reference to somatostatin-producing ones, which often show a “paragaglioma-like” morphology with cells arranged in small tight clusters with sharply outlined polygonal nests (“Zellballen-like”), separated by thin fibrous septa with delicate vasculature [71]. However, it is worth noting that “Zellballen” architecture and paraganglioma-like morphology can be difficult to recognize in small specimens. Both paraganglioma and panNETs with paraganglioma-like morphology show S100-positive sustentacular cells complicating the differential diagnosis, which is mainly based on the lack of low weight cytokeratins positivity and GATA3, tyrosine hydroxylase, PHOX2B, and Hand2 expression in the former [18, 72, 73].

*Composite gangliocytoma/neuroma and neuroendocrine tumor (CoGNET)*, previously known as gangliocytic paraganglioma, is a triphasic tumor composed of neuroendocrine epithelial cells, Schwannian spindle cells, and ganglion cells. Although typically located in the ampulla, rare cases of true primary pancreatic origin have been reported [74–76]. The diagnosis of CoGNET in FNB specimens is difficult because not all three components are typically present simultaneously.

**Fig. 14:**
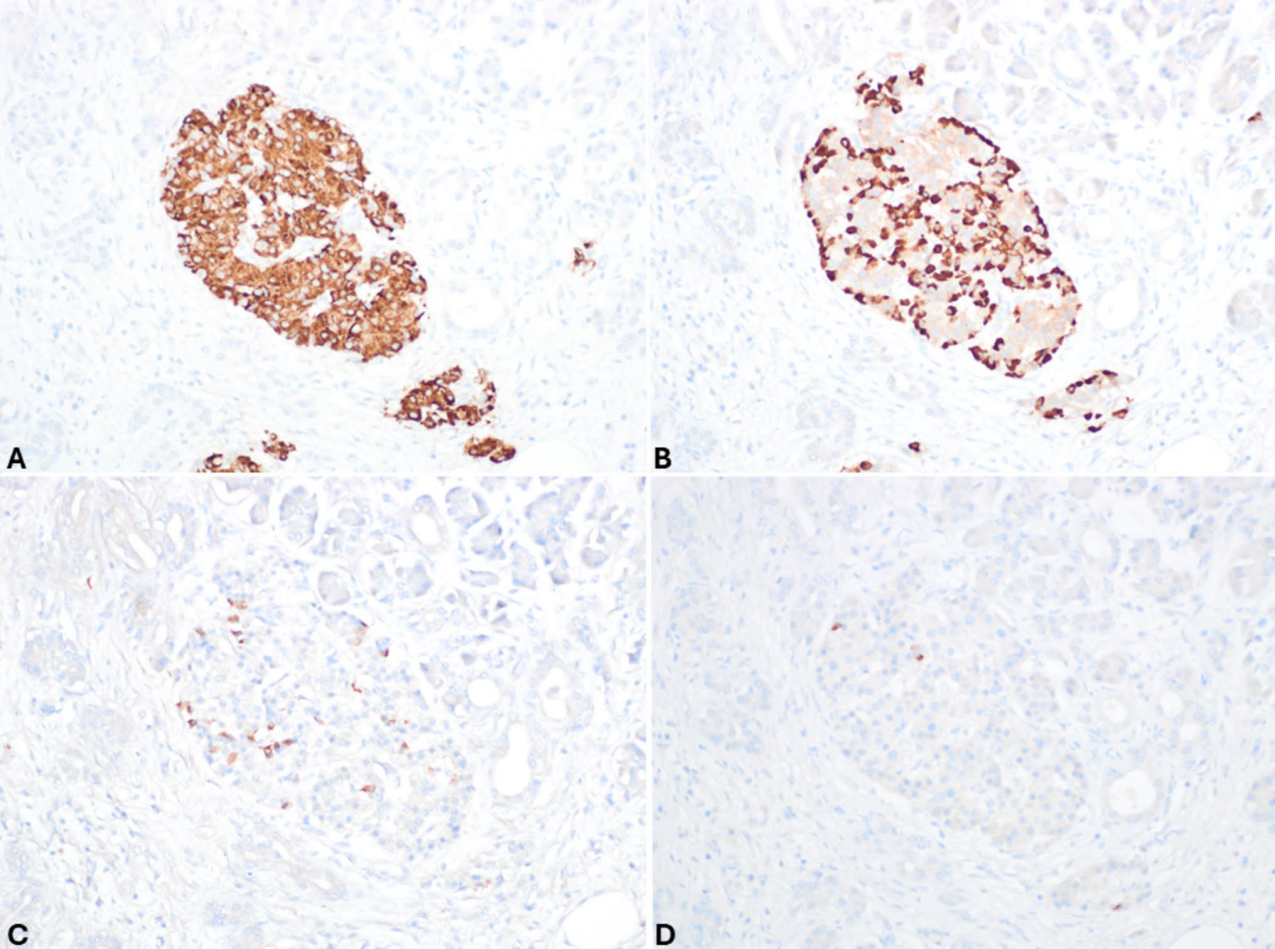
Normal islets show a specific distribution of different neuroendocrine cells with insulin-producing B-cells mainly located in the center (**A**), while glucagon-producing A-cells are at the periphery (**B**). Somatostatin-producing D-cells are rarer and mainly distributed along the periphery (**C**), while pancreatic polypeptide-producing cells (**D**) are rare in the islets with the exception of the posterior lobe of the head where they are numerous (PP lobe)

*Chronic pancreatitis* with islet pseudo-hyperplasia represents an additional issue. Indeed, in these cases, the relative abundance of islets may simulate a panNET, with nested architecture. However, normal/hyperplastic islets are ovoid and, most importantly, show the normal distribution of cells characterized by the central localization of insulin-immunoreactive cells and the peripheral distribution of glucagon-positive and somatostatin-positive cells (Fig. 14). Conversely, neoplastic nests are more irregular, predominantly expressing only one hormone or lacking hormone expression.

### IV) Distinguishing Between Small Cell panNECs and Non-neuroendocrine Mimickers

The morphological features of small cell panNECs are rather specific, but some non-neuroendocrine malignancies may show overlapping morphology.

**Fig. 15:**
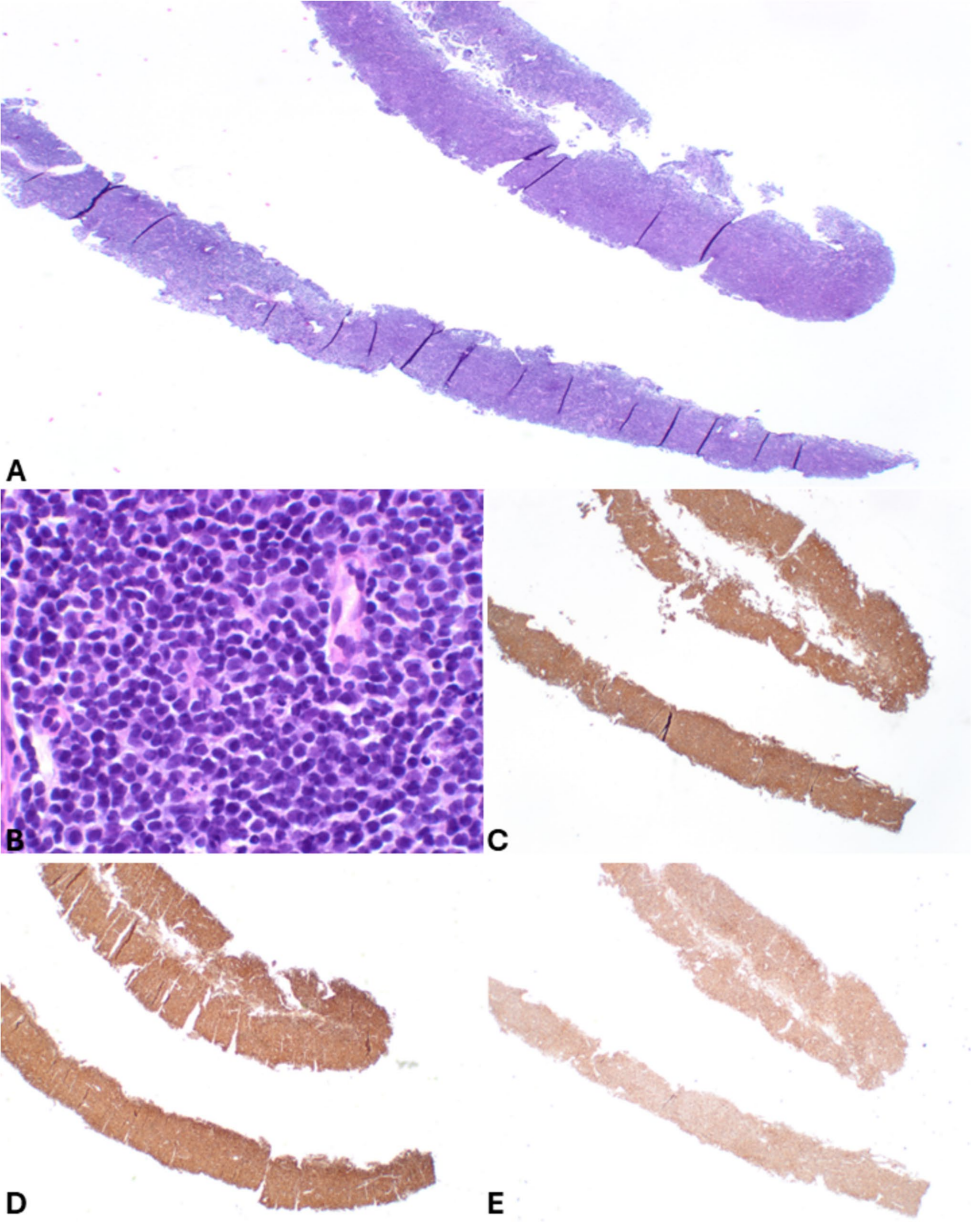
FNB specimen of pancreatic lymphoma, which at low power magnification appears as a “blue tumor” (**A**). At higher magnification (**B**), it is characterized by a diffuse proliferation of small regular cells with elevated N:C ratio, which are strongly positive for CD20 (**C**), CD5 (**D**), and CD23 (**E**). The morphological and immunohistochemical features fit with the diagnosis of small lymphocytic B-cell lymphoma/leukemia

*Non-Hodgkin lymphoma* is one of the possible differential diagnoses. Lymphoma can localize in the pancreas in patients with disseminated disease, while primary non-Hodgkin lymphomas, commonly associated with immunosuppression, are extremely rare, accounting for about 0.5% of pancreatic masses and < 1% of extra-nodal lymphomas [77–79]. Primary pancreatic lymphomas are more frequently represented by diffuse large B-cell lymphoma (77% of cases), followed in decreasing order by follicular lymphoma, Burkitt lymphoma, small lymphocytic B-cell lymphoma, and T-cell lymphoma [78]. Immunohistochemistry is essential for the correct diagnosis and includes the lack of cytokeratin and general neuroendocrine marker expression and positivity for CD45 and other lymphoma markers, depending on the lymphoma type (Fig. 15).

*Ewing sarcoma/primitive neuroectodermal tumor* is a very rare primary pancreatic tumor mainly affecting children or young adults [80]. It is composed of synaptophysin and CD56-positive small round blue cells forming lobules and sheets with frequent molding, necrosis, and high mitotic activity, features also observed in small cell panNECs. For this reason, the differential diagnosis can be problematic. In this context, strong and diffuse membranous immunoreactivity for CD99 observed in Ewing sarcoma is of help [81], but it is worth noting that rare cases of NECs showing CD99 expression have been described [82, 83]. *EWSR1-FLI1* rearrangement is the molecular signature described in pancreatic Ewing sarcoma/primitive neuroectodermal tumor, which immunohistochemically translates into FLI1 nuclear staining [84, 85]. Nuclear immunoreactivity for ERG can be observed in cases with *EWSR1-ERG* fusion [86].

### V) Distinguishing Between Large Cell panNECs and Non-neuroendocrine Mimickers

The solid subtype of *acinar cell carcinoma* (*ACC*) is an insidious mimicker of large cell panNEC because it shares some morphological features including large cells with vesicular nuclei containing prominent eosinophilic nucleoli, abundant eosinophilic cytoplasm, necrosis, and brisk mitotic activity. In addition, as discussed above, general neuroendocrine markers are frequently expressed in ACCs, representing a confounding diagnostic feature. Since the therapy of ACCs and large cell panNECs is different, accurate diagnosis is crucial for clinical management. In this context, every neoplasm showing the histological features described above should be investigated using both acinar and neuroendocrine markers (Table 3). In addition, the search for *BRAF* rearrangement by FISH can be useful since it is found in about 20% of ACCs [87].

*Solid poorly differentiated adenocarcinomas* of the pancreas may resemble large cell panNECs in FNB specimens since they are composed of poorly formed glands with scarce mucin secretion, which can fuse, forming solid areas. The lack of focal expression of neuroendocrine markers and the positivity for CEA (Table 3) and high molecular weight glycoproteins (mucins) are useful to exclude a diagnosis of large cell panNEC.

**Fig. 16:**
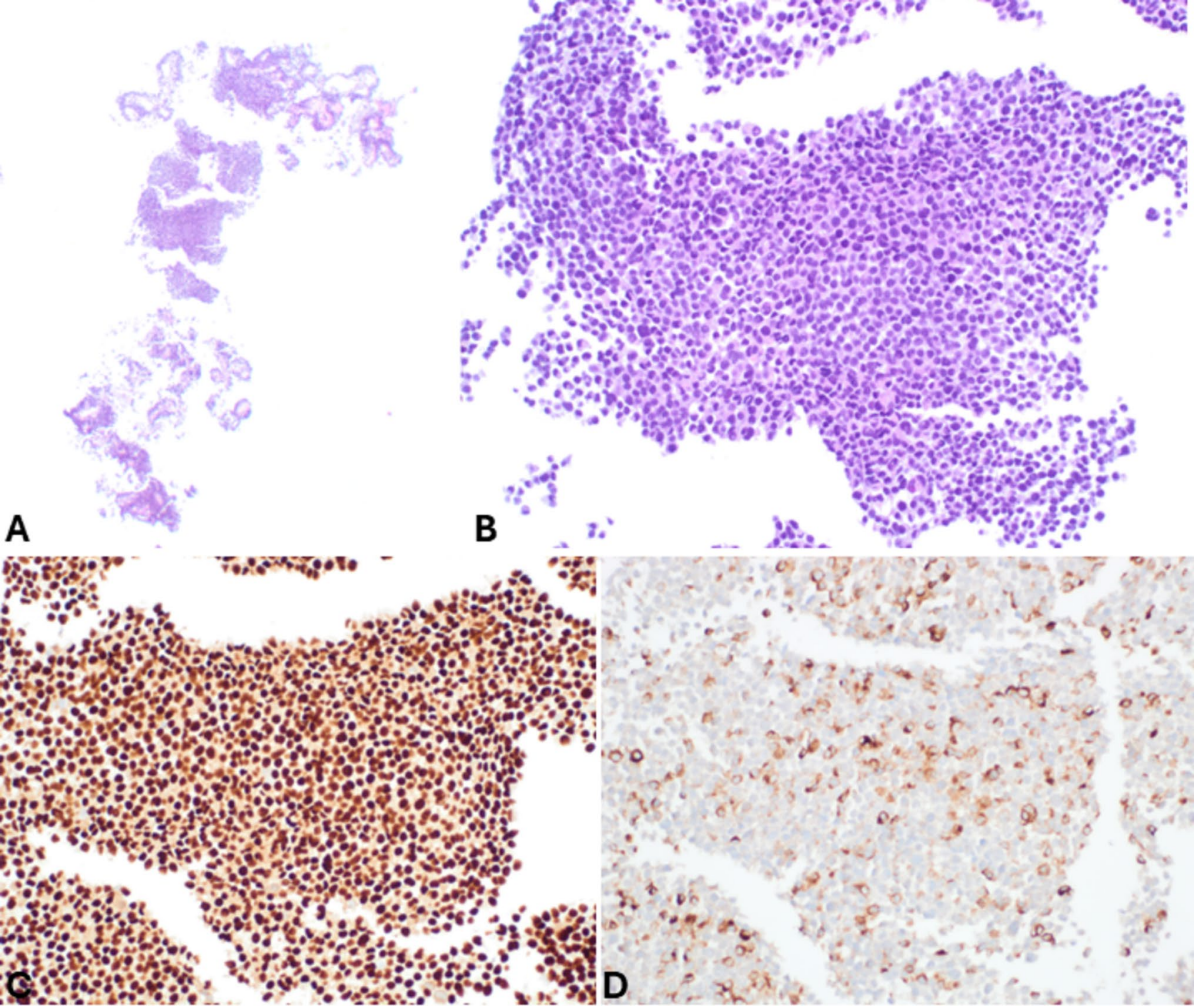
FNB specimen of pancreatic metastasis from cutaneous melanoma (**A**). At higher magnification, it is characterized by a diffuse proliferation of neoplastic cells with eosinophilic cytoplasm and atypical hyperchromic nuclei without desmoplastic stroma (**B**). Tumor cells are diffusely and strongly positive for SOX10 (**C**) and HMB45 (**D**)

*Metastases from melanoma* can be observed in about 2 to 16% of patients with abdominal dissemination from cutaneous melanomas, although metastases from ocular and nasal cavity melanomas have also been reported [88]. Clinical history is fundamental in guiding pathologists, avoiding the unnecessary use of immunohistochemical stains, which may result in a waste of precious material. Tumor cells are large and noncohesive with nuclei frequently showing prominent nucleoli and abundant eosinophilic cytoplasm. Brown pigment, when present, can help the diagnosis, but it is absent in several cases. Immunohistochemistry using SOX10, MART1, PRAME, Human Melanoma Black 45 (HMB-45), and S100 is useful (Table 3 and Fig. 16).

### PanNETs with Specific Morphological Features

In addition to the “conventional” morphological features of panNETs, described above, specific and rarer histological aspects can be observed. When recognized, these features can be indicated in the report since they may be associated with different prognostic features [89]. These “unconventional” features may present challenges for the differential diagnosis with other non-neuroendocrine pancreatic neoplasms.

### Neoplasms with Glandular Structures

**Fig. 17:**
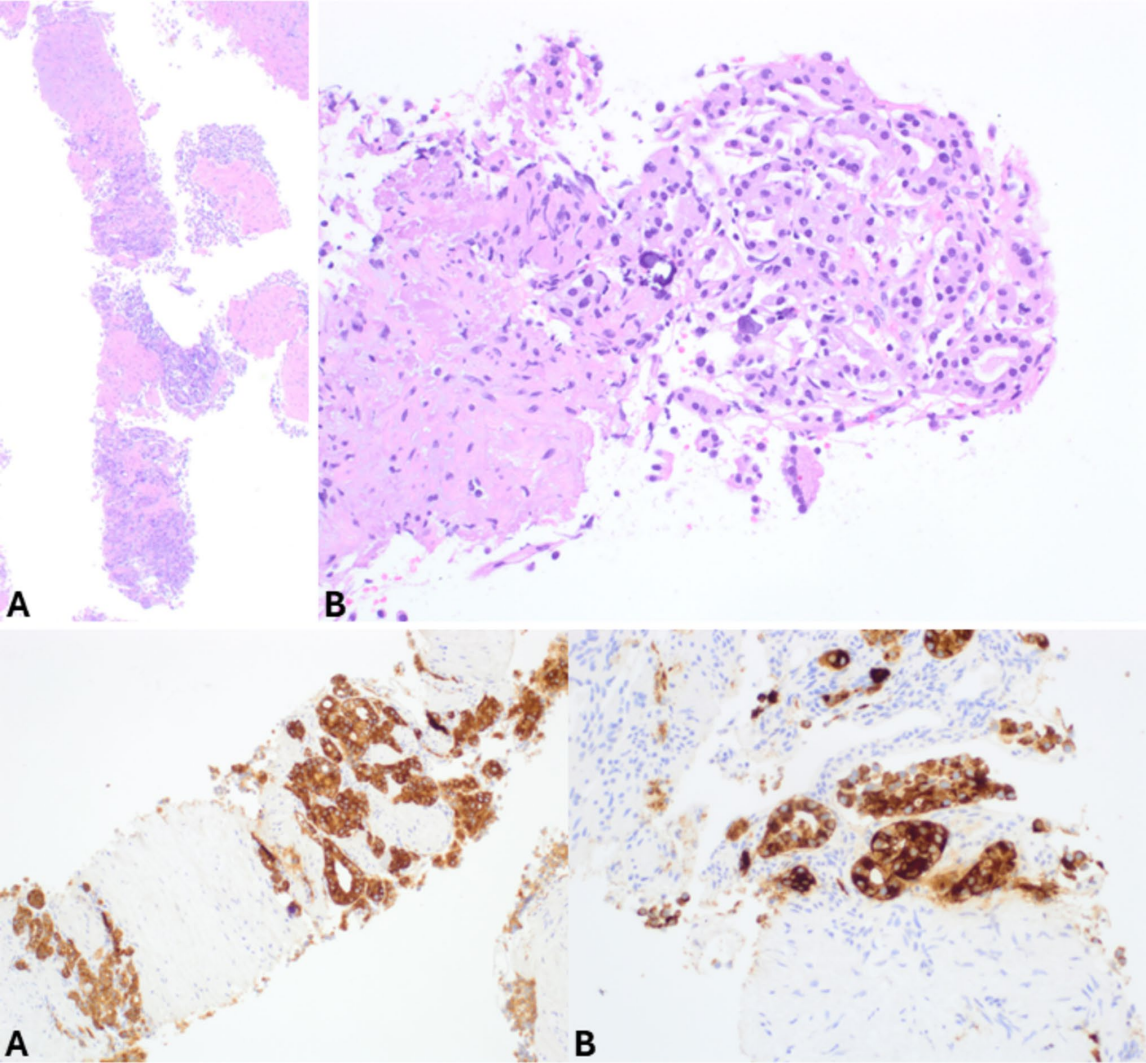
FNB specimen of ampullary somatostatin-producing D-cell tumor infiltrating the pancreatic head (**A**). At higher magnification (**B**), the tumor shows glandular structures composed of well-differentiated cells with round uniform hyperchromic nuclei and relatively abundant eosinophilic cytoplasm. Note the presence of two psammomas. Tumor cells are positive for synaptophysin (**C**) and somatostatin (**D**) (courtesy of dr. Amedeo Sciarra, Histopathology, Central Institute, Valais Hospital, Sion, Switzerland)

**Fig. 18:**
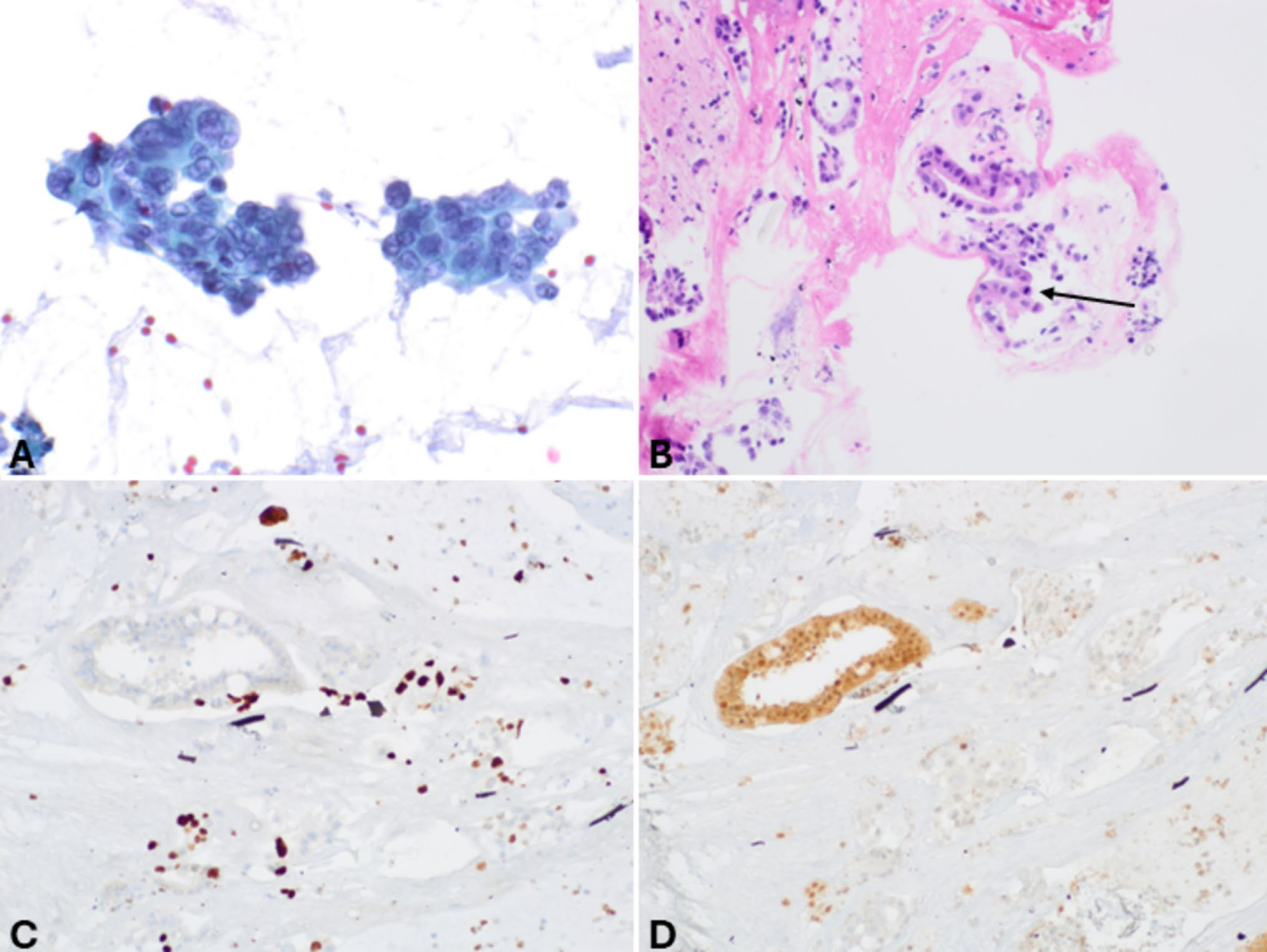
**A** FNA Pap-stained smear of well-differentiated ductal adenocarcinoma showing neoplastic cells with pale nuclei with some indentations and high N:C ratio. **B** The cell block shows atypical neoplastic cells forming irregular glandular structures with an evident mitosis indicated by the arrow. **C** Tumor cells are proliferating as indicated by Ki67 immunoreactivity, which is negative in contaminant intestinal epithelial cells. **D** Tumor cells lack expression of SMAD4/DPC4, which is conversely expressed by contaminant intestinal epithelial cells that serve as internal control

Glandular structures are a usual finding observed in ampullary somatostatin-producing tumors [90], but they can also be rarely observed in panNETs. The presence of glandular structures in FNB specimens (Table 4), especially when obtained from pancreatic head masses, opens at least two differential diagnoses: (i) primary ampullary somatostatin-producing tumor infiltrating the pancreatic head (Fig. 17); and (ii) well-differentiated ductal adenocarcinoma (Fig. 18).

**Table 4:** Differential diagnosis of pancreatic neuroendocrine tumor with glandular aspects

Markers	PanNET	Ampullary somatostatin-producing D-cell NET	Grade 1 PDAC
Psammoma bodies	-/+	+	-
Chromogranin A	+	+, focal	-
Synaptophysin	+	+	-
INSM1	+	+	-
CEA	-	-	+
SMAD4	+	+	-
SSTR5	var	+	-
SSTR2A	+	-	-

*PanNET*, pancreatic neuroendocrine neoplasm; *PDAC G1*, well-differentiated pancreatic ductal adenocarcinoma; *SSTR5*, somatostatin receptor 5; *SSTR2A*, somatostatin receptor 2 A; *var*, variable

Ampullary NETs show a predominant glandular (tubulo-acinar) structure with frequent intraluminal psammoma bodies and only minor trabecular or solid areas. Tumor cells are large with abundant granular eosinophilic to basophilic cytoplasm and regular round nuclei, positive for somatostatin, CK7, MUC1, and SSTR5 and negative for SSTR2A [90]. Chromogranin A expression can be focal. In panNETs, glandular features are more limited and psammoma bodies are rarer [91].

In contrast to gland-forming NETs, well-differentiated ductal adenocarcinomas form glands with much more irregular contours, and tumor cells are positive for CEA and, in about 55–80% of cases, negative for SMAD4/DPC4 [92]. As a rule, when regular, small, bland-looking, and well-formed glands are identified in FNB specimens, the immunohistochemical panel should include CEA, SMAD4, synaptophysin, chromogranin A, and somatostatin to solve the differential diagnostic issue.

### Neoplasms with Oncocytic Features

Cells with oncocytic features characterized by abundant and strongly eosinophilic cytoplasm can be found in both normal and pathological tissues, benign or malignant, and among the latter in either primary or metastatic tumors. The peculiar morphology, due to cytoplasmic accumulation of mitochondria, may be challenging, especially in small samples when crushing artifacts can hide the nuclear features of classical panNETs. In the pancreas, the main differential diagnoses include oncocytic panNETs, oncocytic variants of pancreatic duct adenocarcinomas, intraductal oncocytic papillary neoplasms, SPNs with oncocytic differentiation, and the extremely rare metastasis of renal chromophobe carcinomas [93]. Hence, clinical and radiological correlations are essential for choosing the most appropriate immunohistochemical panel as reported in Table 5. Of note, the oncocytic variant of panNET has been demonstrated to be associated with worse prognosis [89, 94].

**Table 5:** Differential diagnosis of pancreatic neuroendocrine tumors with oncocyte features

Markers	Oncocytic variant panNET	Oncocytic variant PDAC	Oncocytic variant SPN	IOPN	ChRCC
Synaptophysin	+	-	+	-	-
Chromogranin A	+	-	-	-	-
INSM1	+	-	-	-	-
PAX8	- + (if polyclonal)	-	-	-	+
CD117	-	-	-	-	+
CK7, CK19	±	+	-	-	+
CK8, CK18	+	+	±	+	+
Nuclear β catenin	-	-	+	+	-
CD99	-/+	-	+ (dot-like)	-	-

*PanNET*, pancreatic neuroendocrine tumor; *PDAC*, pancreatic ductal adenocarcinoma; *SPN*, solid pseudopapillary neoplasm; *IOPN*, intraductal oncocytic papillary neoplasm; *ChRCC*, chromophobe renal cell carcinoma

### Neoplasms with Clear Cells

The clear aspect of cells can be due to cytoplasmic accumulation of either glycogen (PAS-positive, diastase sensitive) or lipids (which are lost during the standard tissue processing). As for panNETs, clear cell features are related to lipid accumulation and have been described in patients with Von Hippel-Lindau (VHL) and MEN1 syndrome [95, 96], but also in sporadic cases [97]. Neoplastic cells show an optically empty cytoplasm with nuclei losing the classical “salt-and-pepper chromatin.” In the VHL settings, the immunohistochemical reactions for HIF-1a (hypoxia-induced factor 1a), CAIX (carbonic anhydrase IX), and inhibin are positive. The main differential diagnosis in small biopsies includes metastatic clear cell renal cell carcinoma, which is positive for PAX8 and CD10. PAX8 expression may represent a challenging task, because although panNETs are negative when using monoclonal antibodies, they can be positive when using polyclonal antibodies [98]. Other differential diagnoses to be considered include the clear cell variant of pancreatic duct adenocarcinoma (CEA +, SMAD4-), the clear cell variant of SPN (beta-catenin +, LEF1 +, and CD99 +), and neoplasms of the adrenal cortex (cytokeratin -, SF1 +) (Table 6).

**Table 6:** Differential diagnoses of pancreatic neuroendocrine tumors with clear cell features

Markers	Lipid-rich panNET	CCRCC	CC variant PDAC	CC variant SPN	Adrenal cortical tumor
Synaptophysin	+	-	-	+	+
Chromogranin A	+	-	-	-	-
INSM1	+	-	-	-	-
PAX8	+ (if polyclonal)	+	-	-	-
CA IX	+ (in VHL syndrome)	+	-	-	-
CD10	-	+	-	+	±
CK7, CK19	±	-	+	-	-
CK8, CK18	+	+	+	±	-
Nuclear β catenin	-	-	-	+	-
CD99	-/+	-	-	+ (dot-like)	-
SF-1	-	-	-	-	+

*PanNET*, pancreatic neuroendocrine tumor; *CCRCC*, clear cell renal cell carcinoma; *CC variant PDAC*, clear cell variant of pancreatic ductal adenocarcinoma; *CC variant SPN*, clear cell variant of solid pseudopapillary neoplasm; *CA IX*, carbonic anhydrase IX; *VHL*, von Hippel-Lindau

### Neoplasms with Pleomorphic Cells

Pleomorphic cells showing marked “symplastic” (degenerative type) nuclear atypia with multilobated large, bizarre nuclei with smudgy chromatin can be observed in panNETs, but their presence does not have a prognostic impact [99]. However, their identification is a challenge for pathologists since they enter in differential diagnosis with poorly differentiated ductal adenocarcinomas [99] or ACCs with pleomorphic cells [57, 58]. Since in FNB specimens the amount of diagnostic tissue may be limited, other tumor components useful for a definitive diagnosis may be absent. In this context, immunohistochemistry for general neuroendocrine markers, acinar cell markers, and adenocarcinoma markers is essential to solve the diagnostic issue (Table 7).

**Table 7:** Differential diagnosis of pancreatic neuroendocrine tumors with pleomorphic cells

Markers	PanNET	PDAC G3	ACC
Synaptophysin	+	-	-/+
Chromogranin A	+	-	-/+
INSM1	+	-	-
CEA	-	+	-
BCL10	-	-	+
Trypsin	-	-	+

*PanNET*, pancreatic neuroendocrine tumor; *PDAC G3*, poorly differentiated pancreatic ductal adenocarcinoma; *ACC*, acinar cell carcinoma

### Neoplasms with Hyalinized Stroma

Hyalinized stroma can be found in both panNETs and in non-neuroendocrine pancreatic neoplasms showing single or small groups of neoplastic cells arranged in rows or in narrow tubules embedded in a dense sclerosing stroma (Table 8). This aspect is frequently encountered in serotonin-producing NETs, both primary and metastatic, especially from the ileum. As discussed before, ileal serotonin-producing EC-cell NETs show different immunophenotypes with respect to serotonin-producing panNETs. In addition, they seem to induce fibrosis through secretion of different growth factors such as aFGF (acidic fibroblast growth factor) and CTGF (connective tissue growth factor), which are not detected or are produced at low levels in primary serotonin-producing panNETs [67]. Insulinoma is another panNET showing abundant stroma which is composed of amyloid derived from hypersecretion and deposition of amylin (also known as islet amyloid polypeptide—IAPP). The use of Congo red stain and immunoreactivity for insulin facilitates the correct diagnosis in such cases.

**Table 8:** Differential diagnosis of pancreatic neuroendocrine tumors with hyalinized stroma

Markers	5HT panNET	Insulinoma	PDAC
Chromogranin A	+	+	-
Synaptophysin	+	+	-
INSM1	+	+	-
CK7, CK19	±	±	+
CK8, CK18	+	-	+
5HT	+	-	-
Insulin	-	+	-

*5HT*, serotonin; *PanNET*, pancreatic neuroendocrine tumor; *PDAC*, pancreatic ductal adenocarcinoma

## Concluding Remarks

**Fig. 19:**
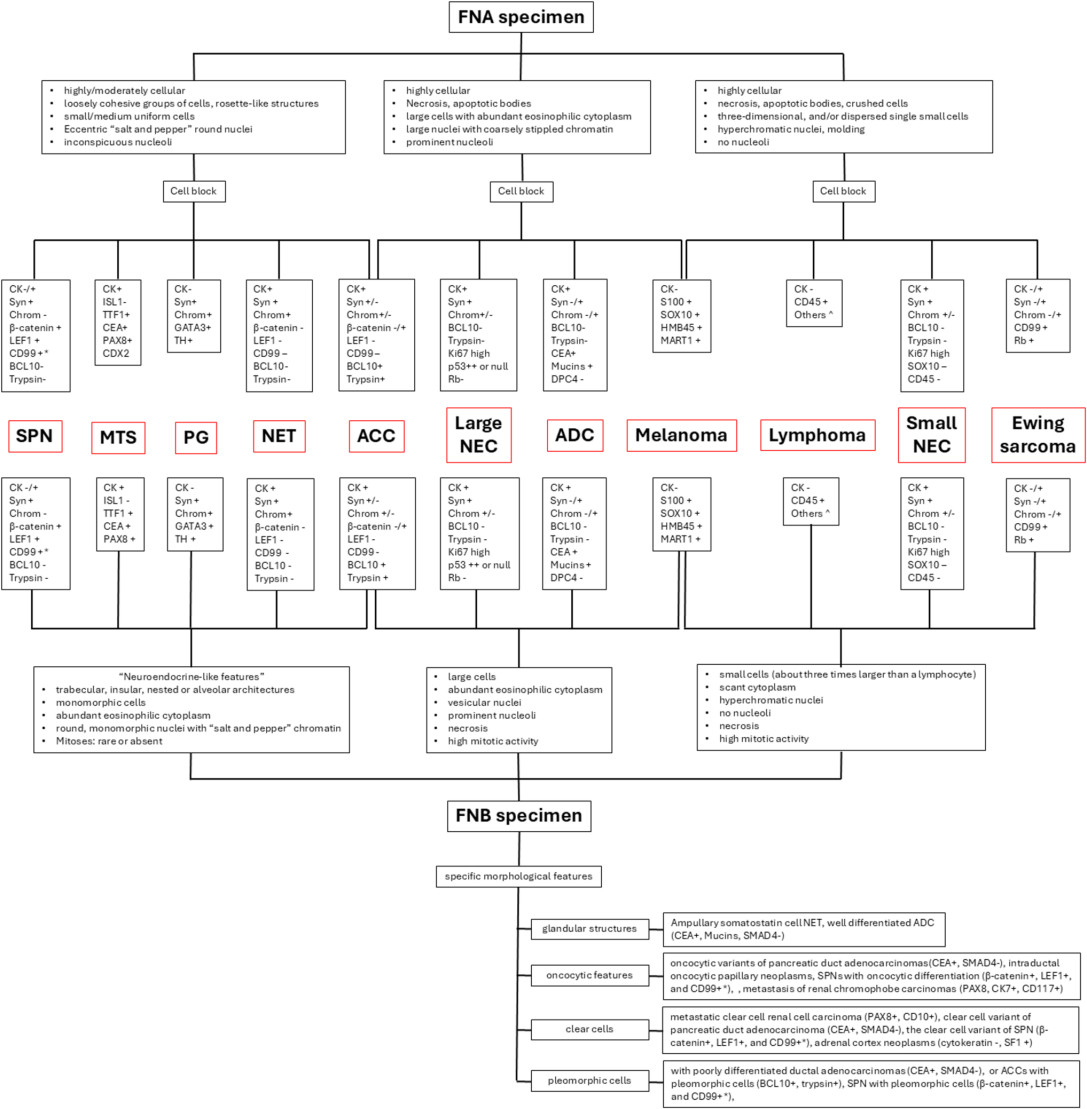
Practical diagnostic algorithm summarizing the work-up of panNEN using FNA or FNB specimens

In the era of interventional radiology, most pancreatic masses are diagnosed using fine needle aspiration (FNA) or fine needle biopsy (FNB). Consequently, pathologists increasingly need to be aware of this reality and adapt to the challenges of using limited cellularity specimens. The choice of a FNA or FNB approach depends on several factors including local practices and the expertise of the local team which includes endoscopists and pathologists. In this paper, we have illustrated the practical approach to the diagnosis of panNENs in both cytological and histological specimens. Morphology plays a crucial and pivotal role in the choice of the most appropriate immunohistochemical tests, with the aim of avoiding wasting precious material. The integration of morphology with immunohistochemistry and, when necessary, molecular analysis facilitates an accurate diagnosis in most cases, enabling the differentiation of panNETs or panNECs from insidious mimickers. A practical diagnostic algorithm summarizing the work-up is illustrated in Fig. 19.

## Data Availability

No datasets were generated or analysed during the current study.
